# Interspecies electron transfer as one of key drivers of methanogenic consortia succession within quorum sensing regulation

**DOI:** 10.1093/ismeco/ycaf165

**Published:** 2025-09-19

**Authors:** Shunan Zhao, Fangzhou Wang, Liuying Song, Shaoqing Zhu, Suo Liu, Kai Zhao, Ruiping Liu, Yu-You Li

**Affiliations:** Center for Water and Ecology, School of Environment, Tsinghua University, Beijing 100084, China; Center for Water and Ecology, School of Environment, Tsinghua University, Beijing 100084, China; College of Environment and Ecology, Taiyuan University of Technology, Taiyuan 030024, China; Center for Water and Ecology, School of Environment, Tsinghua University, Beijing 100084, China; State Key Laboratory of Regional Environment and Sustainability, School of Environment, Tsinghua University, Beijing 100084, China; Center for Water and Ecology, School of Environment, Tsinghua University, Beijing 100084, China; State Key Laboratory of Regional Environment and Sustainability, School of Environment, Tsinghua University, Beijing 100084, China; Center for Water and Ecology, School of Environment, Tsinghua University, Beijing 100084, China; State Key Laboratory of Regional Environment and Sustainability, School of Environment, Tsinghua University, Beijing 100084, China; Department of Civil and Environmental Engineering, Tohoku University, Sendai 9808579, Japan

**Keywords:** quorum sensing, community succession, interspecies electron transfer, syntrophic metabolism, methanogenesis

## Abstract

Robust interspecies interactions are essential for efficient methanogenesis in anaerobic digestion. This study investigated the impact of quorum sensing (QS) enhancement on the succession of methanogenic communities during anaerobic digestion. The QS stimulation via exogenous N-acyl-homoserine lactones enhanced methane production by 18.8%–22.1%. Moreover, QS shaped microbial community succession toward a more deterministic assembly, selectively enriching key syntrophs (*Pelotomaculum*, *Smithella*), and methanogens (*Methanobacterium*, *Methanothrix*). Metagenomic analysis revealed that QS induced genes related to transcription, transport, and cofactor biosynthesis instead of directly regulating carbon metabolism. In this context, interspecies electron transfer emerges as a critical factor regulating interspecies interactions under QS regulation. Specifically, QS enhancement boosted redox mediator secretion, and the concentration of 2-amino-3-carboxy-1,4-naphthoquinone and phenazine increased by 7.8- and 4.8-fold, respectively. QS enhancement also induced higher abundance of c-type cytochromes. Moreover, the higher electron transfer coefficients were detected with 40.2%–89.9% increase. Further, QS also enhanced relative abundance of genes involved in Complex I/III and ferredoxin-dependent hydrogenases, promoting electron flow from syntrophs to methanogens. These effects induced higher relative abundance of genes associated with syntrophic propionate/butyrate oxidation and hydrogenotrophic/acetotrophic methanogenesis. Collectively, given that the similar regulation pathway is widely distributed in anaerobes, these findings identify QS as a critical ecological signal that drives functional microbial succession.

## Introduction

Anaerobic digestion is a mature technology for sustainable waste management and bioenergy production, offering substantial benefits in energy recovery and carbon neutrality [1]. During this process, organic substrates are initially hydrolyzed and fermented into volatile fatty acids (VFAs), including propionate and butyrate [2]. These VFAs are subsequently oxidized into hydrogen and acetate through syntrophic metabolism, which is a critical prerequisite for methanogenesis [2]. The coordinated activity of hydrolytic, fermentative, syntrophic, and methanogenic microorganisms is essential to maintain stable reactor performance [3]. Among these processes, syntrophic metabolism is particularly vital [4], and its inefficiency can lead to VFA accumulation, which is widely recognized as the cause of system failure [5]. However, the thermodynamically unfavorable standard Gibbs free energy of syntrophic metabolism, exemplified by *ΔG_0’_*_propionate-oxidation_ = +76.0 KJ/mol [2, 6], hinders VFA degradation for further methanogenesis (Supplementary Table S1). Only under low hydrogen partial pressure can overcome this inhibition, enabling a negative Gibbs free energy of *ΔG*_propionate-oxidation_ of −15 kJ/mol@1PaH_2_ [7]. Therefore, maintaining effective interactions between syntrophs and methanogens is critical for the efficient and robust operation of anaerobic digesters.

Understanding the mechanisms that govern syntroph–methanogen interactions remain a central topic in anaerobic biotechnology. Interspecies electron transfer (IET) plays a pivotal role in mediating these interactions [8]. Conventional IET occurs via diffusible carriers such as hydrogen and formate, which are transferred from syntrophs to methanogens [3, 9, 10]. More recently, direct interspecies electron transfer (DIET) has gained attention as an alternative pathway, wherein electrons are transferred directly through electrically conductive pili (nanowires) or c-type cytochromes [11, 12]. DIET has been shown to overcome the thermodynamic limitations imposed by hydrogen accumulation, thereby enhancing methanogenesis. [13]. To promote DIET, researchers have tried to incorporate conductive materials into digester or use ethanol as electron donor [14, 15]. Beyond these established approaches, quorum sensing (QS) has emerged as a potentially novel strategy for modulating syntrophic interactions [16].

The QS pathway is a microbial communication system regulated by signaling molecules, including N-acyl-homoserine lactones (AHLs), autoinducing peptides, and pheromones [17]. This pathway is widespread in anaerobic digesters, with AHLs being the most well-recognized signaling molecules [18]. Previous studies have demonstrated that QS can influence methanogenic performance and restructure microbial communities [19, 20]. Network analyses further suggest that syntrophs and methanogens are closely associated with QS signaling molecules in digesters [21]. Our earlier research showed that QS stimulation selectively enriched *Geobacter* and *Methanothrix*, enhancing DIET-mediated methanogenesis [16]. Besides *Geobacter*, other syntrophs, such as *Pelotomaculum* and *Smithella*, are more commonly observed in digesters [3], and also possess QS-related genes, indicating broader potential responsiveness to QS regulation [22, 23]. These findings inspire us that QS may serve as a universal mechanism to mediate syntroph–methanogen interactions. Nevertheless, current studies primarily focus on community abundance changes and rarely quantify the extent to which QS selectively enriches key functional microbes. Moreover, the drivers of QS-induced community restructuring remain poorly understood.

In this study, we investigated the response of methanogenic consortia to QS stimulation during two representative syntrophic metabolisms (propionate and butyrate oxidation) to elucidate the directional succession and underlying mechanisms of QS regulation. Using 16S rRNA gene amplicon sequencing, we assessed microbial community succession, co-occurrence patterns, and assembly processes to infer the directionality of community shifts under QS regulation. Metagenomic analysis was employed to identify key functional genes involved in syntrophic metabolism and interspecies electron transfer. Electron flux calculations, extracellular polymeric substance (EPS) characterization, and redox mediators’ measurements were integrated to clarify the IET capacity. This study provides new insight into how QS influences community structure and function through modulation of IET processes, thereby offering a promising strategy to modulate methanogenic consortia for efficient methanogenesis.

## Method and materials

### Substance, inoculum, and reagents

The anaerobic granular sludge, sourced from a full-scale mesophilic digester, was used to treat citrate wastewater. The sludge had a total solids content of 12.3% ± 0.2% and a volatile solids content of 9.1% ± 0.2%. As two kinds of frequently detected signal molecules in digesters, C4-HSL (CAS: 67605-85-0) and C6-HSL (CAS: 147852-83-3) were employed as exogenous inducers to potentiate QS activity [16, 24, 25].

### Detailed operational conditions of the AnSBR system

It is known that high concentrations of propionate and butyrate can have an impact on the pH of the solution within the system, and propanol and butanol have the ability to be converted into propionate and butyrate, which can then be further utilized for methanogenesis [26]. Therefore, propanol and butanol were used as alternatives to propionate, and the detailed components are presented in Supplementary Table S2. The dose and application method of the exogenous C4-HSL and C6-HSL were consistent with those in previous studies [16, 27].

Specifically, two sets of 500-ml working-volume AnSBRs were operated in parallel for 76 days under mesophilic conditions (37 ± 0.2°C, maintained by a water bath). Each reactor was stirred at 150 rpm (BPC Instruments, Sweden). Initially, 400 ml of synthetic feedstock was loaded into each reactor, followed by a 5-min nitrogen purge. Subsequently, 100 g of inoculated sludge was added, followed by a second 20-min nitrogen purge. The initial chemical oxygen demand (COD) of the feedstock was 5000 mg/l, and the organic loading rate (OLR) was maintained at 1 g COD/l·d. Based on different degradation kinetics of the two alcohols and previous operating experience, substrate-specific loading conditions were adjusted accordingly (Supplementary Table S3). The hydraulic retention time ranged from 5 to 10 days. To mitigate acidification stress, NaHCO₃ was added as a buffering agent (Supplementary Fig. S1).

### Environmental microorganism data collection

Raw microbial sequencing data were retrieved from the National Center for Biotechnology Information database, encompassing 63 samples from anaerobic digesters, bioelectrochemical systems, anammox reactors, and other systems. Details of the datasets and method are summarized in Supplementary Table S4 and Text S1. All sequences were processed separately using the same workflow to minimize technical bias from different projects.

### Analytical methods

The samples were filtered through 0.22 μm syringe filters and stored at 4°C prior to analysis. Total solids and volatile solids were measured using standard methods [28], and biogas was collected using gas sampling bags [16]. The concentrations of VFAs were determined by high-performance liquid chromatography (Waters, USA). The pH values were measured using a digital pH meter (Thermo, USA), and COD was assessed using commercial test kits (HACH, USA). EPSs, including loosely bound (L-EPS) and tightly bound (T-EPS), were extracted via heat extraction (Text S2) [29]. As protein is the main electroactive substance in EPS, the protein content was determined using the BCA Protein Assay Kit (Beijing Boxbio Science & Technology, China). After batch tests, redox mediators such as 2-amino-3-carboxy-1,4-naphthoquinone (ACNQ), flavin adenine dinucleotide (FAD), and phenazine were quantified (Text S3) [30]. The sludge was rinsed in PBS to eliminate growth medium and stained with FITC (Beijing Solarbio Science & Technology, China) for protein visualization of the EPS [31]. Particle size distribution was measured using a laser particle size analyzer (Beckman, USA). Electron transfer capacity of the microbial consortia was evaluated using cyclic voltammetry (CV) (Chenhua, China), as detailed in Text S4. Throughout the digestion process, 10 ml microbial samples were collected at each stage and stored at −80°C for microbial community and metagenomics analysis. The detailed method is presented in Text S5.

### Statistical analysis

All experiments were conducted in triplicate and results were presented as mean ± SD. One-way analysis of variance was performed to evaluate statistical significance, with the designation of “significant(ly)” being strictly reserved for results demonstrating statistical significance (*P*-value < .05). The modified Gompertz equation was used for kinetics analysis (Text S6). The R-project was used to conduct Mantel test, network, and microbial community assembly analysis by “linkET,” “igraph,” and “NST” package (Text S7) [32, 33]. Electron transfer flux for interspecies hydrogen transfer (IHT) was calculated based on Fick’s Law (Text S8) [14, 34].

## Results and discussion

### Positive performances of methanogenic digestion

**Figure 1:**
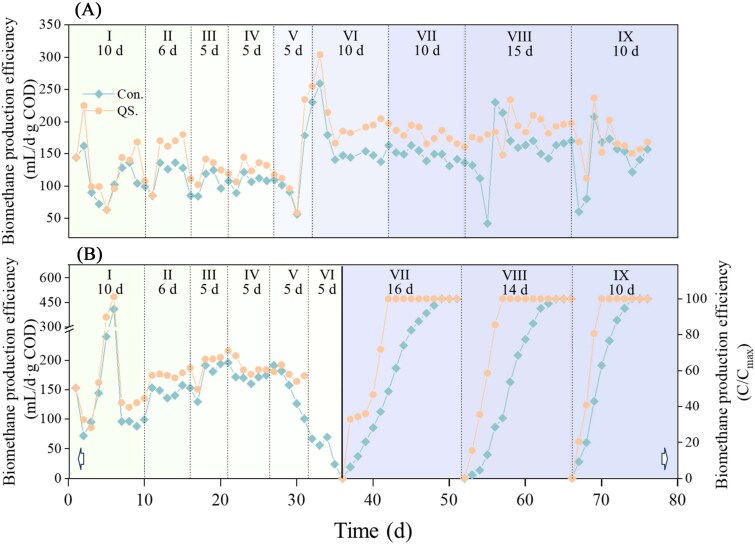
The biomethane production performance of propanol-fed systems (A) and butanol-fed systems (B). Note: The detailed organic load was exhibited in Supplementary Table S3.

Generally, QS enhancement improved methanogenesis efficiency by 22.1% and 18.8% in propanol- and butanol-fed systems (Fig. 1). Two sets of systems exhibited similar CH_4_ content (*P*-value > .1) (Supplementary Fig. S2), indicating that QS enhancement primarily improved yield rather than gas composition. Specifically, the average biomethane production increased by 17.6%–25.9% during Stage I–IX of the propanol-fed systems and by 9.5%–24.4% during Stage I–V of the butanol-fed systems (Supplementary Fig. S3). Under batch-fed conditions, the maximum gas production rate in butanol-fed systems increased by 41.3%, 99.4%, and 78.0% during Stages VII–IX, respectively (Supplementary Fig. S4). Notably, although the metabolic activity of the system increased after acclimation, the QS enhancement system still maintained a higher metabolic activity. Similarly, the biogas production rate in propanol-fed systems was 37.2% faster on Day 75 (Supplementary Fig. S4).

All reactors maintained stable operation, and pH levels remained within an optimal range (6.9–7.6) under lower OLRs, regardless of QS signal molecule addition (Supplementary Fig. S1). Under elevated OLR conditions, QS enhancement systems exhibited reduced VFAs accumulation and lower effluent COD, indicating more efficient syntrophic metabolism (Supplementary Fig. S5). Specifically, with QS enhancement, the average effluent COD concentration of propanol-fed system decreased by 46.5% and 61.1% at Stages VII and VIII, respectively (Supplementary Fig. S5). Similarly, butanol-fed systems also observed lower VFAs concentration at Stage I–IV within AHLs introduction (Supplementary Fig. S6). The QS enhancement system exhibited robust process stability when subjected to excessive organic loading rate (ORL) shocks at Stage V–VI. Interestingly, although elevated VFAs were observed in the QS enhancement system during fed-batch fermentation (Stages VII), they were rapidly consumed, accompanied by a marked increase in methane production (Fig. 1 and Supplementary Fig. S6). This dynamic suggests that QS enhancement promoted stronger syntrophic metabolism, allowing for efficient VFAs degradation and sustained methanogenic activity.

### Quorum sensing modulates succession, assembly, and network interactions of methanogenic consortia

#### Evolution of microbial communities during the enrichment process

**Figure 2:**
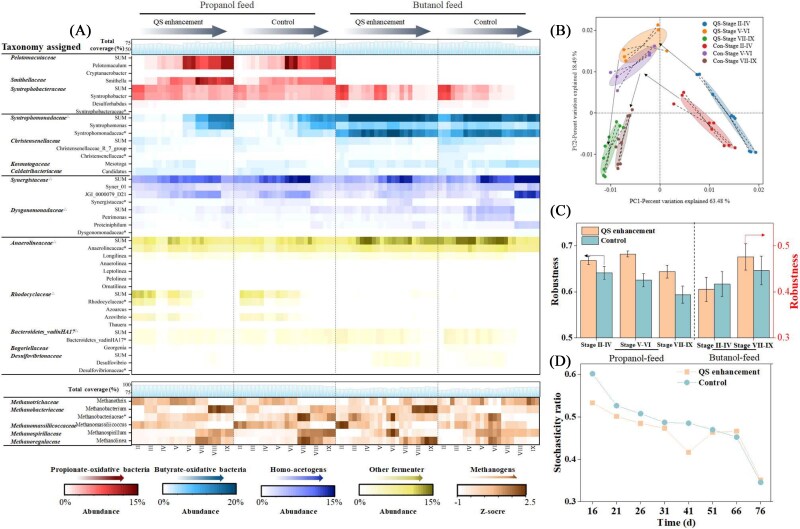
Microbial community structure and assembly within QS regulation. Taxonomic compositions (A); PCA analysis in propanol-fed systems (B); robustness of two sets of systems (C); stochasticity ratio changes in propanol-fed systems (D).

As shown in Fig. 2, syntrophs and methanogens progressively dominated the microbial community throughout the digestion process. In propanol-fed systems, detailed taxonomic analysis revealed *Pelotomaculum*, *Syntrophobacter*, and *Smithella* as the dominant syntrophic propionate-oxidizing bacteria (SPOB). Their abundance markedly increased during Stages VI–IX (Supplementary Fig. S7), corresponding to enhanced syntrophic metabolism and elevated methane yields [35]. Specifically, *Pelotomaculum* increased by 12.7%, 5.3%, and 42.6% in Stages VII–IX, respectively, while *Smithella* showed an average increase of 49.8% under QS enhancement during Stages V–IX. Notably, *Smithella* can dismutate propionate into acetate and butyrate [36], facilitating the enrichment of *Syntrophomonas* (Supplementary Fig. S8), a key syntrophic butyrate-oxidizing bacterium (SBOB), which was conductive to butyrate degradation. As for butanol-fed systems, *Syntrophomonas* was seen as the dominant SBOB. QS regulation notably enhanced its abundance by 18.7%–51.9% during Stages II–VI (Fig. 2). Although syntrophic propionate-oxidizing bacteria were also detected in these systems, their abundance gradually declined over time, suggesting their initial presence likely originated from the inoculum (Fig. 2).

Besides, QS regulation also influenced other microbial populations relevant to or competing with methanogenic metabolism. The syntrophy process produced abundant acetate and hydrogen for methane production. In these digesters, *Methanothrix* was the predominant methanogen for acetotrophic methanogenesis and was enriched with QS enhancement, as reported in previous studies [16, 37]. Conversely, elevated acetate levels from increased alcohol loading also promoted the enrichment of *Synergistaceae*, syntrophic acetate-oxidizing bacteria (SAOB). However, the enhanced presence of *Methanothrix* under QS regulation suppressed *Synergistaceae* abundance, reflecting a stronger acetoclastic pathway. Besides, the higher ORL caused more IET flux, favoring hydrogenotrophic methanogenesis. This was reflected in a marked increase in *Methanobacterium* abundance, from 17.7%–19.6% in Stage II to 28.8%–35.7% in Stage VIII, highlighting a shift in methanogenic pathways toward hydrogenotrophic methanogenesis [3, 38, 39]. This transition highlights the IHT process between syntrophs and methanogens in the syntrophy-methanogenesis process [38, 39]. For propanol-fed systems, QS enhancement significantly enriched *Methanobacterium*, with its relative abundance rising by 23.9%–124.1% (Fig. 2). Interestingly, in butanol-fed systems, *Methanobacterium* was more abundant in the Control, possibly due to DIET, wherein *Methanothrix* accepts electrons from SBOB [40, 41].

#### Quorum sensing also affecting the direction of microbial succession

In propanol-fed systems, principal coordinate analysis revealed distinct clustering of microbial communities based on both QS presence and OLR levels, suggesting that QS and OLR jointly directed microbial succession (Fig. 2 and Supplementary Fig. S9). Alpha diversity indices, including Chao1, Shannon, and Simpson, were consistently lower in QS enhancement systems (Supplementary Fig. S10). Negative correlations between Shannon diversity and OLR further indicated that increasing substrate loading and QS regulation led to selective enrichment of functionally important taxa, such as *Pelotomaculaceae* and *Smithellaceae* (Supplementary Fig. S10).

To investigate microbial interactions, we constructed 10 co-occurrence networks for Control and QS enhancement communities across both systems (Supplementary Fig. S11). QS enhancement system enhanced networks exhibited fewer nodes but more edges. Moreover, the key microbes had more connectivity to other nodes in QS enhancement communities, indicating that QS selectively enriched key functional microbes and enhanced interspecies connectivity (Supplementary Fig. S12) [42]. This was accompanied by more robust and cohesive community structures (Fig. 2 and Supplementary Fig. S12), suggesting that QS fostered stronger cooperative interactions.

We further evaluated community assembly mechanisms, distinguishing between deterministic and stochastic processes [43]. A diminished role of stochastic processes was observed in the QS enhancement system, indicated by lower stochastic ratio (ST ratio), modified stochasticity ratio (MST) ratio, and *R*^2^ value in the neutral community model (Fig. 2 and Supplementary Fig. S13). The results corroborated the advantages of deterministic processes (ST < 0.5) for the almost community construction (Fig. 2). Moreover, deterministic processes dominated the between-group assembly, increasing from 39.9%–46.7% (Stage II) to 64.8%–65.5% (Stage IX) for propanol-fed systems, which further supported the conclusion that QS regulation exerts selective pressure that shapes community composition through targeted microbial interactions [44].

### Acclimation of methanogenic consortia to quorum sensing

#### Key response genes identification and proposed regulatory mechanism

**Figure 3:**
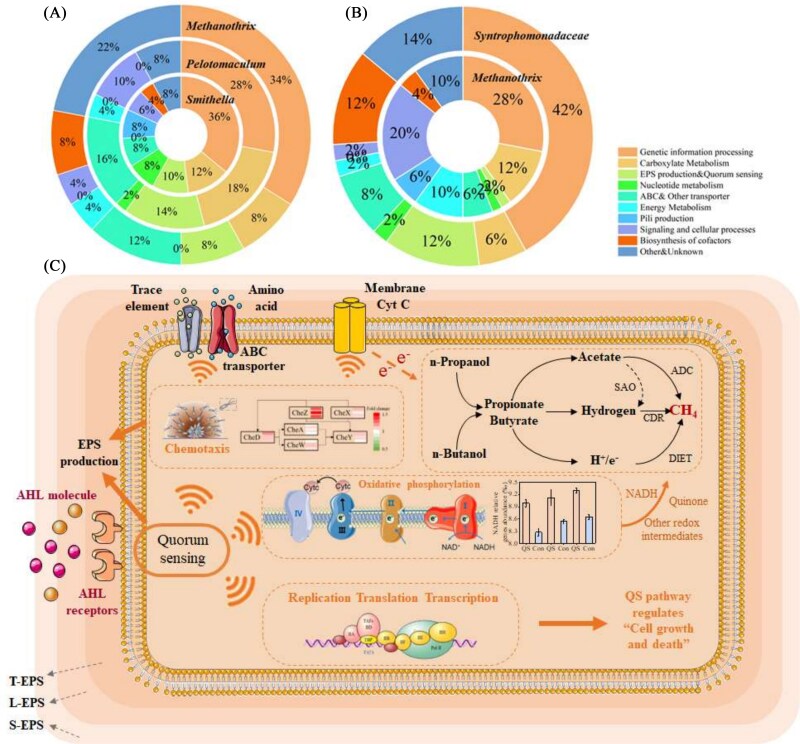
The top 50 up-regulated high-abundance genes of key functional genus in propanol-fed systems (A) and butanol-fed systems (B); and the gene regulatory interaction diagram (C) based on key genes. Note: The key functional genus is defined as the highest enrichment genus in Section 3.2.1. High-abundance genes are defined as those in the top 50% of overall abundance.

Microbial ecology suggests that QS governs community succession through deterministic processes. However, the specific microbial functions regulated by QS and their impact on community structure remain unclear. According to previous studies, we focused on identifying genes highly expressed in responsive microbial strains, which may directly correspond to QS regulation (Fig. 3) [45, 46]. Contrary to expectations that carbon metabolism, central to syntrophy and methanogenesis, would dominate gene abundance increase, our results revealed that genes involved in “genetic information processing,” “nucleotide metabolism,” “ABC and other transporters,” and “cofactor biosynthesis” were more significantly enriched (Fig. 3).

To elucidate the regulatory network underlying QS responses, we analyzed gene expression patterns of candidate key genes (Fig. 3). The addition of exogenous AHLs significantly increased the abundance of QS-related pathways by 12.6%–16.4% in propanol-fed and 10.3%–15.7% in butanol-fed systems (Supplementary Fig. S14). QS enhancement modulated transcription and translation processes, evidenced by numerous increases or decreases in gene abundance (Supplementary Fig. S15), and increased the abundance of chemotaxis-related genes. Mantel tests confirmed a strong correlation between chemotaxis and QS pathways (Supplementary Fig. S16). This coordinated increase likely stimulated ABC transporter proteins critical for amino acid and trace element uptake, particularly genes from the liv and cbi families (Supplementary Table S5). The increased influx of amino acids and trace elements provides essential substrates for the biosynthesis of coenzymes and membrane proteins, which are essential for efficient methanogenesis and EPS production [46]. Furthermore, QS and chemotaxis pathways co-regulate biofilm formation, facilitating close physical interactions between syntrophs and methanogens for stronger IET processes [14, 47]. Together, these findings suggest that QS does not directly alter the core metabolic capacity of individual microorganisms but modulates their metabolic interactions, particularly by influencing IET between syntrophs and methanogens.

#### The characteristics of interspecies electron transfer

**Figure 4:**
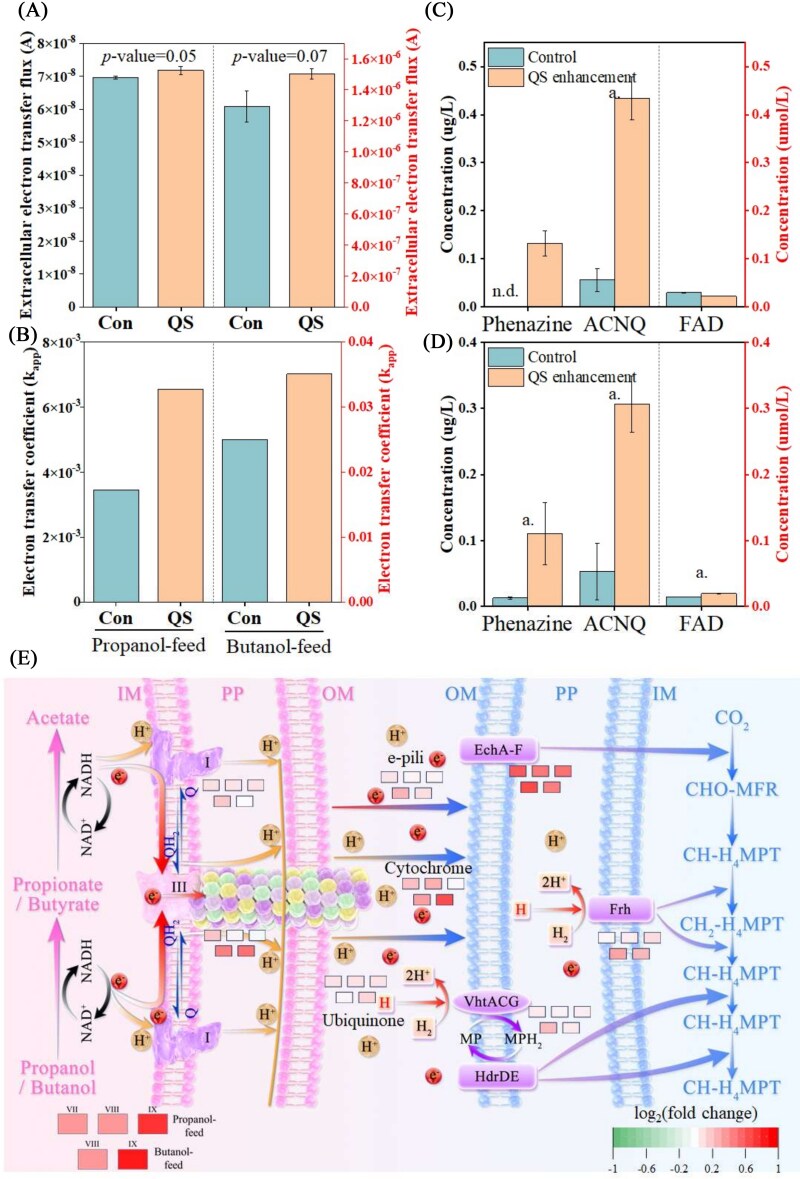
The electron transfer fluxes (A) and electron transfer coefficient (B) in two sets of system at Stage VII. The concentration of typical redox mediator in propanol-fed systems (C) and butanol-fed systems (D). The diagram of electron transfer process (E). Note: ACNQ means 2-amino-3-carboxy-1,4-naphthoquinone. FAD means flavin adenine dinucleotide. The a. means the *P*-value < .01. The n.d means: no detection. Fold change was defined as data divided by average of control. Thus, the control had a 1-fold change, its log_2_(FC) is about zero. The depth of the color blocks represents the strength of gene variation abundance. Different positions indicate changes in different systems at various stages.

In the traditional model of IET, electrons are transferred from syntrophs to methanogens primarily via diffusion of intermediates such as hydrogen and formate [12, 48]. Despite QS enhancement, both QS enhancement and Control exhibited similar granule formation, likely driven by hydraulic shear forces during stirring [49]. Consequently, maximum IHT fluxes were comparable in both systems (Fig. 4). Notably, two distinct redox peaks at −0.10 V and 0.07 V were detected in the propanol-fed system (Supplementary Fig. S17), corresponding to the electron transfer activities of c-type and a-/d-type outer membrane cytochromes [50]. The presence of these electroactive proteins suggests that DIET may also occur in this system [51]. The homogeneous spatial distribution of *Methanothrix* and syntrophic bacteria further supports this cooperative interaction (Supplementary Fig. S18).

Conceptually, DIET involves in three components of (i) electron generation by syntrophs, (ii) extracellular electron transfer (EET), and (iii) electron acceptance by methanogens [10, 52]. The increased intensity of redox peaks in the QS enhancement system indicates higher EET capacity (Supplementary Fig. S17) [53], with electron transfer coefficients increasing by 89.9% and 40.2% in propanol- and butanol-fed systems, respectively (Fig. 4). The *RegAB* is representative to detect redox signals and regulate electron transfer systems, such as *Pet ABC* and *CycA*, via a two-component system (Supplementary Fig. S19). Genes related to both electrical signaling (*RegAB*) and EET components (*c*-Cyts) showed higher abundance in QS enhancement system. Then, the abundance of genes encoding EET components, including conductive pili, *c*-Cyts, ubiquinone, and other redox mediators, was analyzed (Supplementary Table S6). Two sets of systems exhibited similar abundance of genes related to conductive pili secretion (Supplementary Table S6), which confirmed the low abundance of *Geobacter* (Supplementary Fig. S20). In contrast, genes for c-type cytochromes and ubiquinone biosynthesis were significantly enriched (*P*-value < .01) in the QS enhancement (Supplementary Table S6), corroborating the CV redox peak data. Similarly, genes associated with ubiquinone biosynthesis were also enriched in the QS enhancement system (Supplementary Table S6).

To identify key contributors to enhanced EET, we measured extracellular concentrations of redox mediators, including ACNQ, FAD, and phenazine. QS enhancement significantly increased ACNQ and phenazine production, while FAD levels remained unchanged (Fig. 4). Previous studies identified ACNQ, FAD, and phenazine as typical redox mediators in *Aeromonas hydrophila*, *Shewanella oneidensis*, and *Pseudomonas aeruginosa*, respectively [30, 54–56]. QS can regulate their secretion of these mediators via AHL signaling molecules [54, 55, 57, 58]. Although these genera were not dominant here, other microbes likely respond to QS by secreting these redox compounds. However, the different behaviors of these redox mediators might result from variations in dominant microbial populations, and the underlying mechanisms require further investigation.

The pronounced redox peaks observed in CV curves indicate the presence of electroactive proteins (Supplementary Fig. S17). EPS harbors electroactive substances (such as flavins and c-type cytochromes), and stronger DIET systems were characterized by higher EPS production [59]. Consistent with this, QS enhancement stimulated EPS production, with protein content in T-EPS and L-EPS increasing by 90.6% and 38.3%, respectively (Supplementary Fig. S21). Moreover, the FITC staining further confirmed elevated protein levels (Supplementary Fig. S22). The EEM spectra revealed increased dissolved organic matter in EPS, including tyrosine-, tryptophan-, and microbial byproduct-like substances (Supplementary Fig. S23) [60, 61], compounds correlated with enhanced methanogenesis and DIET [62]. Moreover, the EPS extracted from QS enhancement system exhibited stronger charge quantity, as indicated by the larger CV curve area, further confirming higher DIET capability (Supplementary Fig. S24).

The stronger electron transfer capability also implies better electron-producing ability in syntrophs and electron-accepting ability in methanogens (Fig. 4). For syntrophs, nicotinamide adenine dinucleotide (NAD^+^) accepted H^+^ and e^−^ generated from the oxidation of organic substrates to form NAD-H [63], which subsequently transfers electrons through Complexes I and III before shuttling them to methanogens via EET [15]. In this process, corresponding genes were highly expressed in both propanol- and butanol-fed systems (Supplementary Table S7). Similarly, methanogens accepted electrons by ferredoxin-dependent hydrogenase (Ech), F_420_-reducing hydrogenase, and heterodisulfide reductases [64–67]. With the introduction of AHLs, the abundance of these genes significantly improved (Supplementary Table S8). For example, the abundance of Ech was increased by 56.3%, 43.9%, and 46.5% in Stages VII–IX of propanol-fed systems, respectively. Additionally, the fluorescent component associated with F_420_ (excitation wavelength = 420 nm), a coenzyme involved in methanogenesis [60], exhibited higher intensity in the QS enhancement system (Supplementary Fig. S23). These findings imply that the QS enhancement significantly improves electron transfer capability, particularly the DIET pathway.

#### Higher gene abundance of syntrophic and methanogenic pathways

**Figure 5:**
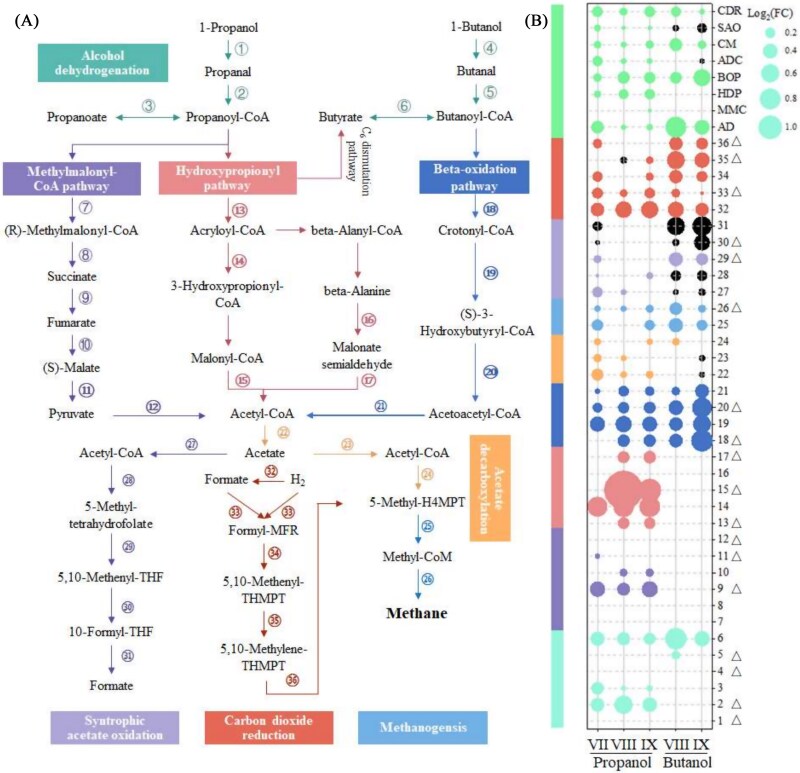
Proposed pathways for n-propanol-feed methane production (A) and key functional genes abundance changes in digestion process based on metagenomics analysis (B). Note: The bubble chart of key functional genes abundance involved in alcohol degradation, MMC, HDP pathway, BOP, SAO, carbon dioxide reduction, acetate decarboxylation, and common methanogensis based on log_2_(fold change). Results exhibit the independent triplicates. “△” symbol represents redox intermediates participated reactions (Table S9).

To evaluate the impact of QS-enhanced electron transfer on syntrophic metabolism and methanogenesis, we compared the abundance of key functional genes in both propanol- and butanol-fed systems (Fig. 5). Notably, genes encoding alcohol dehydrogenases showed significant increase of 21.7% at Stage VII in propanol-fed system (*P*-value < .01), and of 31.5%–69.5% at Stages VIII and IX in butanol-fed system. In particular, propanal dehydrogenase, which oxidizes propanal, producing electrons that reduce NAD^+^ to NAD-H via ubiquinone reductase (Enzyme commission, EC 7.1.1.2), increased by 25.5%, 51.0%, and 30.1% at Stages VII to IX [63]. Similarly, butanal dehydrogenase (EC 1.2.1.57), responsible for NAD-H generation during butanol oxidation, exhibited a 10.7%–15.7% increase. Corresponding increases in ubiquinone reductase gene abundance further supported enhanced electron transfer capacity (Supplementary Table S6 and Supplementary Table S7).

**Figure 6:**
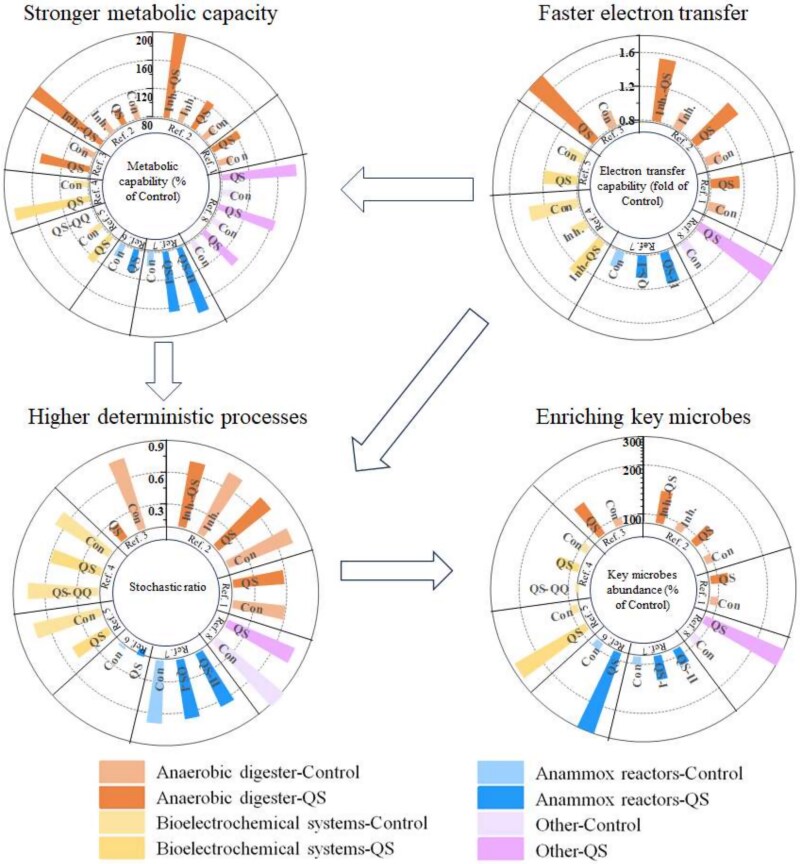
The relationship of metabolic capability, electron transfer, community assembly, and microbial succession in anaerobic digesters, bioelectrochemical systems, anammox reactors, and other environments. Note: Detailed references exhibited in Supplementary Table S4.

The methylmalonyl-CoA (MMC), hydroxypropionyl (HDP), and beta-oxidation (BOP) pathways, essential for syntrophic oxidation of propionate and butyrate, also exhibited elevated gene abundances. [2, 68]. For the MMC pathway, the higher gene abundance was primarily ascribed to the stronger conversion of succinate to fumarate in Step-9, which involves redox intermediates such as NAD-H and quinones. In syntrophic metabolism, redox intermediates of NAD/NAD-H, flavin adenine dinucleotide (FAD)/FAD-H_2_, and Fd_ox_/Fd_red_ have been reported to play crucial roles in these processes for electron transfer [63, 69]. The abundance of the genes involved in these redox intermediates was more pronounced upon QS enhancement, which might be relative to the higher electron transfer capability. With regard to the HDP pathway, nearly all genes of HDP pathway showed higher abundance levels, and the total abundance was increased by 7.2%, 12.9%, and 14.6% in propanol-fed systems at Stages VII, VIII, and IX, respectively. QS enhancement also increased the abundance of key genes with redox intermediates participating, including EC: 1.2.1.18 and EC: 1.3.8.1 (Fig. 5). Correspondingly, QS enhancement also improved the abundance of genes for butyrate degradation (BOP pathway).

Methanogens tend to assimilate H_2_/formate and acetate from syntrophs, and convert them into 5-methyl-THMPT for further methane production [70]. With QS enhancement, the overall abundance of methanogenesis genes increased by 6.5%–12.6% and 3.4%–13.6% in propanol- and butanol-fed systems (Fig. 5). Specially, hydrogenotrophic methanogenesis genes exhibited 9.2%–16.0% increases in the propanol-fed system, likely reflecting enhanced IET capacity. The role of *Methanothrix*, a key DIET methanogen, was supported by its active participation (Supplementary Fig. S25) [8]. Exogenous AHL addition also induced the acetate decarboxylation pathway genes, promoting acetoclastic methanogenesis while suppressing syntrophic acetate oxidation (SAO), as evidenced by decreased SAO gene abundance. Notably, since several genes in this pathway are critical components of other metabolic networks, an elevated abundance at step 29 of the SAO (also serving for C1-unit interconversion) pathway does not necessarily indicate enhanced SAO activity. Consequently, the abundance of genes involved in the common methanogenesis pathway from 5-methyl-THMPT to methane increased by 5.0%–10.2% and 10.5%–22.9% in propanol- and butanol-fed systems, respectively, upon QS enhancement. Notably, methyl-CoM reductase (EC 2.8.4.1), a crucial enzyme potentially accepting electrons via EET [67], showed the highest relative increase (9.0%–24.4%), underscoring the importance of electron transfer in methanogenesis. Overall, QS-induced enhancement of IET significantly accelerates electron-dependent reactions, thereby intensifying syntrophic metabolism and methane production.

#### Link among quorum sensing, interspecies electron transfer, and community composition

In summary, QS strongly influences microbial community structure by regulating IET pathways. As a modulatory signal, QS seldom directly alters the metabolic capacities but subtly steers their metabolic interactions, indirectly modifies their metabolic capability, and induces community succession. This broad regulatory effect explains extensive role of QS in environmental biotechnology applications [71]. To further elucidate the relationships among QS, IET, and community assembly, we analyzed sequencing data from anaerobic digesters, bioelectrochemical systems, anammox reactors, and other environments. Generally, there is a strong correlation among these indicators (Supplementary Table S10). Exogenous addition of AHLs enhanced QS pathways, which increased the expression of genes encoding electron transfer components such as c-type cytochromes and redox mediators, thereby strengthening DIET capability (Fig. 6). On one hand, QS may stimulate key microbes, including syntrophs and methanogens, to secrete electroactive substances, akin to phenazine synthesis regulation in *P. aeruginosa* PA14 [72]. On the other hand, it is postulated that the markedly enhanced electron transfer is largely driven by “public goods cheats,” who reap the benefits of the shared resource without contributing their fair share [73]. This suggests that these redox mediators do not exclusively originate from the secretions of these key microbes, but may also be derived from other microbes [74]. This “free-rider” phenomenon endows these key microbes with enhanced competitive advantages [17]. Such strengthened interspecific cooperation drives greater metabolic activity and promotes community succession through increased deterministic assembly processes (Fig. 6).

Specifically, within the context of QS enhancement, hydrogenotrophic methanogens and *Methanothrix* would harvest electrons from IET, especially DIET, thereby reducing the hydrogen partial pressure in the system. This reduction favors syntrophic oxidation of propionate and butyrate, which is further accelerated by rapid electron transfer. These conditions enrich syntrophic bacteria such as *Pelotomaculum* and *Smithella*, reflected in increased syntrophic gene abundance. Additionally, QS stimulation enhances acetoclastic methanogen activity, leading to rapid proliferation of *Methanothrix* and increased acetate consumption. This competitive acetate uptake suppresses *Synergistaceae* growth. Conversely, in Control with higher SAOB, excess hydrogen accumulates, inhibiting propionate oxidation. Together, QS-induced enhancement of IET facilitates electron flow from syntrophs to methanogens, promoting syntrophy and methanogenesis, and enabling these functional microbes to outcompete other microbes such as SAOB and sulfate-reducing bacteria.

### Implications

Our study demonstrates that QS regulation significantly enhances methanogenesis efficiency by promoting IET capabilities, thereby driving the succession of methanogenic consortia. The positive outcomes provide a theoretical basis for manipulating microbial communities in anaerobic digestion systems to improve biogas production and process stability. Except for industrial methanogenesis processes, QS is also widespread in natural settings such as sediments, rice paddies, and ruminants [71], which are major natural methane emission sources for climate change. Exploring the relationship between QS and methanogenic consortia offers new strategies for developing targeted bioagents to curb methane emissions, like adding them to feed for controlling ruminal methane, thus contributing to the Sustainable Development Goals.

Actually, QS enhancement and quenching have been widely applied in various environmental processes, such as anammox [75], activated sludge process [76], nitrification and denitrification [77], CO_2_ reduction [78], and chain elongation [79]. These processes demonstrate that QS regulation can significantly influence microbial community structure and enrich key functional microbes through the introduction of exogenous QS molecules and specific secreting strains. Notably, the introduction of specific secreting strains seldom enriches the corresponding secreting populations [80]. Of course, different environmental processes exhibited divergent succession direction within QS regulation. For example, QS enhancement preferentially enriched methanogens in digesters, whereas it disproportionately accumulates denitrifiers in anoxic systems [16, 81]. Generally, the beneficial effects observed in strain regulation stem from regulatory mechanisms that mirror those activated by exogenous QS molecules (Fig. 6). Based on these meaningful results, this study provides the proof of concept for widely enriching syntrophs through QS enhancement, and demonstrates that QS regulation can augment deterministic processes within the microbiota for targeted enrichment. It is plausible that the mechanisms underlying community succession processes, as well as the genetic regulation elucidated in this study, are also relevant to microbial succession and responses in other environmental contexts. The data generated in our study have the potential to be incorporated into other studies assessing the interaction between microbial succession mechanisms and QS enhancement and quenching in soils and other environmental matrices. Future research should incorporate advanced microbial ecology theories, such as network modeling and community assembly analysis, alongside next-generation sequencing and -omics technologies, to further elucidate the regulatory mechanisms of microbial communities under QS influence. Such studies should facilitate the development of function-based microbial strains or consortia tailored for bioremediation and biotechnology in waste and wastewater treatment applications.

## Supplementary Material

Supporting_Information_ycaf165

## Data Availability

The data generated or analyzed during this study are included in this published article and its supplementary information files.
