# Supplementary material for: Interspecies electron transfer as one of key drivers of methanogenic consortia succession within quorum sensing regulation
Source: ISME Commun. 2025 Sep 19;5(1):ycaf165. doi: 10.1093/ismeco/ycaf165 (PMC12503159; doi:10.1093/ismeco/ycaf165)
Supplement: Supporting_Information_ycaf165 [file supporting_information_ycaf165.docx]

Supplementary material for:

Interspecies electron transfer as one of key drivers of methanogenic consortia succession within quorum sensing regulation

Shunan Zhao^a^, Fangzhou Wang^a^, Liuying Song^b^, Shaoqing Zhu^a^, Suo Liu^c^, Kai Zhao^a,c^, Ruiping Liu^a,c^[[1]](#footnote-1)^*^, Yu-You Li^d^

1. Center for Water and Ecology, School of Environment, Tsinghua University, Beijing 100084, China
2. College of Environment and Ecology, Taiyuan University of Technology, Taiyuan 030024, China
3. State Key Laboratory of Regional Environment and Sustainability, School of Environment, Tsinghua University, Beijing 100084, China
4. Department of Civil and Environmental Engineering, Tohoku University, Sendai 9808579, Japan

The Supporting Information contains 56 pages, 7 texts, 25 figures, 10 tables.

# Table S1 The energy performance of hydrogen production and consumption.

| Substance | Reaction | δG（KJ/mol） |
| --- | --- | --- |
| Ethanol | CH_3_CH_2_OH+H_2_O=CH_3_COO^-^+H^+^+2H_2_ | +9.6 |
| Butyrate | CH_3_CH_2_CH_2_COO^-^+2H_2_O=2CH_3_COO^-^+H^+^+2H_2_ | +48.5 |
|  | CH_3_CH_2_CH_2_COO^-^+2HCO_3_^−^=2CH_3_COO^−^+H^+^+2HCOO^−^ | +45.5 |
| Propionate | CH_3_CH_2_COO^−^+ 3H_2_O=CH_3_COO^−^+ HCO_3_^−^+ H^+^+3H_2_ | +76.0 |
|  | CH_3_CH_2_COO^−^+2HCO_3_^−^=CH_3_COO^−^+3HCOO^−^+ H^+^ | +72.4 |
|  | CH_3_CH_2_COO^−^+2H_2_O=CH_3_COO^−^+CO_2_+3H_2_ | +72.0 |
| Hydroxyacetic acid | CH_2_OHCOO^-^+H^+^+H_2_O=2CO_2_+3H_2_ | +19.3 |
| Aromatic compound | C_6_H_5_COO^-^+6H_2_O=3CH_3_COO^−^+2H^+^+CO_2_+3H_2_ | +49.5 |
|  | C_6_H_5_OH+5H_2_O=3CH_3_COO^−^+3H^+^+2H_2_ | +10.2 |
| Amino acid | CH_3_CH(NH_3_^+^)COO^-^+2H_2_O= CH_3_COO^−^+CO_2_+2H_2_+NH_4_^+^ | +2.7 |
| Hydrogen consumption reaction | 4H_2_+2CO_2_= CH_3_COO^−^+H^+^+2H_2_O | -94.9 |
|  | 4H_2_+CO_2_=CH_4_+2H_2_O | -131.0 |

# Table S2 The composition of the feedstocks.

| Ingredient | Concentration (mg/L) |
| --- | --- |
| CaCl_2_·2H_2_O | 50 |
| MgCl_2_·6H_2_O | 200 |
| NH_4_Cl | 500 |
| KH_2_PO_4_ | 110 |
| K_2_HPO_4_ | 170 |
| Na_2_SO_4_ | 50 |
| Wolfe's Vitamin Solution | 1 mL |
| Wolfe's Mineral Solution | 1 mL |
| Propanol/Butanol | 5-50 gCOD/L^a^ |
| NaHCO_3_ | 2000 |

Note: a. The propanol/butanol loading was changed at different stage.

# Table S3 The influent organic matter content and change of organic loading rate (OLR) in the two sets of reactors throughout the whole experiments.

|  | Propanol-feed | | | | Butanol-feed | | | |
| --- | --- | --- | --- | --- | --- | --- | --- | --- |
|  | During (days) | Influent COD (mg/L) | HRT (d) | ORL (Kg COD/m^3^·d) | During (days) | Influent COD (mg/L) | HRT (d) | ORL (Kg COD/m^3^·d) |
| Stage I | 10 | 5000 | 5 | 1 | 10 | 5000 | 5 | 1 |
| Stage II | 6 | 10000 | 10 | 1 | 6 | 10000 | 5 | 2 |
| Stage III | 5 | 15000 | 10 | 1.5 | 5 | 20000 | 5 | 4 |
| Stage IV | 5 | 20000 | 10 | 2 | 5 | 25000 | 5 | 5 |
| Stage V | 5 | 30000 | 10 | 3 | 5 | 35000 | 5 | 7 |
| Stage VI | 10 | 25000 | 10 | 2.5 | 5 | 0 | - | 0 |
| Stage VII | 10 | 30000 | 10 | 3 | 16 | - | - | 10 g COD dosage |
| Stage VIII | 15 | 40000 | 10 | 4 | 14 | - | - | 10 g COD dosage |
| Stage IX | 10 | 50000 | 10 | 5 | 10 | - | - | 10 g COD dosage |

# Table S4 The detailed information of collected data from previous studies

| Groups | NCBI | Samples Number | System type | Reference |
| --- | --- | --- | --- | --- |
| 1 | PRJNA1096298 | 4 | Anaerobic digester | (Lv et al 2024) |
| 2 | PRJNA1185413 | 4 | Anaerobic digester | (Huang et al 2025) |
| 3 | - | 6 | Anaerobic digester | (Zhao et al 2024b) |
| 4 | PRJNA868509 | 24 | Bioelectrochemical systems | (Cheng et al 2023) |
| 5 | SRR17798820-SRR17798822 | 3 | Bioelectrochemical systems | (Zhu et al 2023) |
| 6 | PRJNA979122 | 4 | Anammox reactor | (Zhang et al 2024) |
| 7 | PRJNA1166751 | 3 | Anammox reactor | (Yang et al 2025) |
| 8 | PRJNA1078137 | 15 | Other | (Ping et al 2024) |

Note: For microbial samples that were not subjected to replicate testing, stochastic tests were performed by triplicating the data of sample to provide a preliminary assessment of stochastic ratio.

# Table S5 The abundance and description of typical ABC transporters related genes in control and QS enhancement systems.

| - | | | | | | K_ID | | | | Description | | Propanol-feed | | | | | | | Butanol-feed | | | |  |  |  |  |  |  |  |
| --- | --- | --- | --- | --- | --- | --- | --- | --- | --- | --- | --- | --- | --- | --- | --- | --- | --- | --- | --- | --- | --- | --- | --- | --- | --- | --- | --- | --- | --- |
|  |  |  |  |  |  |  | | |  | | | | Stage VII | | | Stage VIII | | | Stage IV | | Stage VIII | | | Stage IV | |  |  |  |  |
|  |  |  |  |  |  |  |  |  |  | QS | | | | Con | | QS | | Con | | QS | | Con | | QS | Con | | QS | Con | |
| Trace metals transport | | | | | K15584 | | | | nickel transport system substrate-binding protein | | | 1.61E-06 | 1.99E-06 | | 3.83E-07 | 3.18E-06 | 1.13E-06 | 1.44E-06 | 1.99E-06 | 2.29E-06 | 2.87E-06 | 3.53E-06 |  |  |  |  |  |  |  |
|  |  |  |  |  | K15585 | | | | nickel transport system permease protein | | | 1.81E-06 | 4.44E-06 | | 6.06E-07 | 3.48E-06 | 3.57E-07 | 3.10E-06 | 1.01E-06 | 2.01E-06 | 2.46E-06 | 2.94E-06 |  |  |  |  |  |  |  |
|  |  |  |  |  | K15586 | | | | nickel transport system permease protein | | | 0.00E+00 | 6.04E-06 | | 0.00E+00 | 4.59E-06 | 2.68E-07 | 1.84E-06 | 1.27E-06 | 3.38E-06 | 1.17E-06 | 4.16E-06 |  |  |  |  |  |  |  |
|  |  |  |  |  | K15587 | | | | nickel transport system ATP-binding protein | | | 0.00E+00 | 3.25E-06 | | 0.00E+00 | 2.04E-06 | 0.00E+00 | 1.94E-07 | 7.20E-07 | 3.02E-06 | 3.34E-06 | 2.76E-06 |  |  |  |  |  |  |  |
|  |  |  |  |  | K10824 | | | | nickel transport system ATP-binding protein | | | 0.00E+00 | 2.88E-06 | | 0.00E+00 | 2.35E-06 | 0.00E+00 | 7.69E-07 | 1.05E-06 | 2.90E-06 | 1.90E-06 | 2.23E-06 |  |  |  |  |  |  |  |
|  |  |  |  |  | K02006 | | | | cobalt/nickel transport system ATP-binding protein | | | 6.17E-04 | 5.44E-04 | | 6.96E-04 | 6.63E-04 | 7.04E-04 | 6.57E-04 | 8.31E-04 | 7.47E-04 | 8.91E-04 | 7.65E-04 |  |  |  |  |  |  |  |
|  |  |  |  |  | K02007 | | | | cobalt/nickel transport system permease protein | | | 7.53E-04 | 6.77E-04 | | 8.77E-04 | 8.45E-04 | 9.21E-04 | 8.10E-04 | 1.05E-03 | 1.07E-03 | 1.18E-03 | 1.07E-03 |  |  |  |  |  |  |  |
|  |  |  |  |  | K02008 | | | | cobalt/nickel transport system permease protein | | | 6.12E-04 | 5.47E-04 | | 7.01E-04 | 6.61E-04 | 7.01E-04 | 6.44E-04 | 7.72E-04 | 7.41E-04 | 8.92E-04 | 7.41E-04 |  |  |  |  |  |  |  |
|  |  |  |  |  | K02009 | | | | cobalt/nickel transport protein | | | 3.31E-04 | 2.70E-04 | | 4.12E-04 | 3.86E-04 | 4.10E-04 | 3.40E-04 | 3.99E-04 | 4.29E-04 | 4.39E-04 | 4.11E-04 |  |  |  |  |  |  |  |
|  |  |  |  |  | K16915 | | | | nickel transport protein | | | 1.86E-04 | 1.24E-04 | | 1.50E-04 | 1.44E-04 | 1.68E-04 | 1.42E-04 | 2.22E-04 | 2.79E-04 | 2.10E-04 | 2.57E-04 |  |  |  |  |  |  |  |
|  |  |  |  |  | SUM | | | | | | | 2.50E-03 | 2.18E-03 | | 2.84E-03 | 2.71E-03 | 2.91E-03 | 2.60E-03 | 3.28E-03 | 3.28E-03 | 3.62E-03 | 3.26E-03 |  |  |  |  |  |  |  |
| Sulfate transport | | | | | K02048 | | | | sulfate transport system substrate-binding protein | | | 2.75E-05 | 2.05E-05 | | 2.57E-05 | 1.31E-05 | 2.92E-05 | 1.93E-05 | 1.12E-05 | 1.38E-05 | 7.71E-06 | 1.07E-05 |  |  |  |  |  |  |  |
|  |  |  |  |  | K02046 | | | | sulfate transport system permease protein | | | 1.33E-05 | 1.26E-05 | | 1.47E-05 | 7.67E-06 | 1.59E-05 | 9.45E-06 | 6.48E-06 | 7.76E-06 | 4.49E-06 | 5.95E-06 |  |  |  |  |  |  |  |
|  |  |  |  |  | K02045 | | | | sulfate transport system ATP-binding protein | | | 3.82E-05 | 3.13E-05 | | 3.24E-05 | 2.66E-05 | 4.16E-05 | 3.66E-05 | 8.53E-06 | 1.08E-05 | 6.02E-06 | 1.04E-05 |  |  |  |  |  |  |  |
|  |  |  |  |  | K02047 | | | | sulfate transport system permease protein | | | 1.59E-05 | 1.29E-05 | | 1.57E-05 | 5.62E-06 | 1.69E-05 | 9.73E-06 | 6.30E-06 | 1.02E-05 | 3.94E-06 | 6.76E-06 |  |  |  |  |  |  |  |
|  |  |  |  |  | SUM | | | | | | | 9.49E-05 | 7.73E-05 | | 8.85E-05 | 5.30E-05 | 1.04E-04 | 7.51E-05 | 3.26E-05 | 4.25E-05 | 2.22E-05 | 3.38E-05 |  |  |  |  |  |  |  |
| amino acid transport | | | | | K01995 | | | | branched-chain amino acid transport system ATP-binding protein | | | 1.38E-03 | 1.21E-03 | | 1.38E-03 | 1.29E-03 | 1.49E-03 | 1.35E-03 | 1.13E-03 | 9.86E-04 | 1.17E-03 | 9.87E-04 |  |  |  |  |  |  |  |
|  |  |  |  |  | K01996 | | | | branched-chain amino acid transport system ATP-binding protein | | | 1.34E-03 | 1.20E-03 | | 1.34E-03 | 1.26E-03 | 1.52E-03 | 1.34E-03 | 1.13E-03 | 1.04E-03 | 1.14E-03 | 1.01E-03 |  |  |  |  |  |  |  |
|  |  |  |  |  | K01997 | | | | branched-chain amino acid transport system permease protein | | | 1.30E-03 | 1.18E-03 | | 1.30E-03 | 1.24E-03 | 1.39E-03 | 1.26E-03 | 1.16E-03 | 9.95E-04 | 1.21E-03 | 9.60E-04 |  |  |  |  |  |  |  |
|  |  |  |  |  | K01998 | | | | branched-chain amino acid transport system permease protein | | | 1.41E-03 | 1.26E-03 | | 1.41E-03 | 1.40E-03 | 1.55E-03 | 1.42E-03 | 1.25E-03 | 1.09E-03 | 1.30E-03 | 1.06E-03 |  |  |  |  |  |  |  |
|  |  |  |  |  | K01999 | | | | branched-chain amino acid transport system substrate-binding protein | | | 1.91E-03 | 1.71E-03 | | 1.91E-03 | 1.73E-03 | 1.88E-03 | 1.76E-03 | 1.75E-03 | 1.71E-03 | 1.80E-03 | 1.65E-03 |  |  |  |  |  |  |  |
|  |  |  |  |  | SUM | | | | | | | 7.36E-03 | 6.56E-03 | | 7.36E-03 | 6.91E-03 | 7.84E-03 | 7.14E-03 | 6.41E-03 | 5.81E-03 | 6.62E-03 | 5.67E-03 |  |  |  |  |  |  |  |

# Table S6 The abundance and description redox intermediates related genes in control and QS enhancement systems.

|  | K_ID | Propanol-feed | | | | | | Butanol-feed | | | |
| --- | --- | --- | --- | --- | --- | --- | --- | --- | --- | --- | --- |
|  |  | Stage VII | | Stage VIII | | Stage IX | | Stage VIII | | Stage IX | |
|  |  | QS | Con | QS | Con | QS | Con | QS | Con | QS | Con |
| e-pili | K02650 | 1.30E-04 | 1.16E-04 | 1.74E-04 | 1.72E-04 | 1.58E-04 | 1.66E-04 | 1.73E-04 | 1.10E-04 | 1.92E-04 | 1.46E-04 |
|  | K02653 | 6.00E-04 | 5.46E-04 | 6.02E-04 | 5.89E-04 | 5.50E-04 | 5.34E-04 | 4.18E-04 | 3.67E-04 | 4.54E-04 | 4.68E-04 |
|  | K02652 | 7.22E-04 | 5.97E-04 | 6.39E-04 | 7.09E-04 | 5.84E-04 | 6.51E-04 | 6.86E-04 | 4.87E-04 | 7.85E-04 | 6.16E-04 |
|  | K02662 | 3.70E-04 | 3.52E-04 | 4.06E-04 | 3.93E-04 | 3.83E-04 | 3.63E-04 | 2.67E-04 | 2.38E-04 | 2.90E-04 | 2.79E-04 |
|  | K02666 | 2.10E-04 | 2.34E-04 | 2.41E-04 | 1.93E-04 | 2.60E-04 | 2.02E-04 | 7.73E-05 | 1.11E-04 | 7.98E-05 | 1.02E-04 |
|  | K02663 | 8.35E-05 | 8.04E-05 | 9.11E-05 | 7.94E-05 | 9.20E-05 | 7.65E-05 | 3.95E-05 | 1.25E-05 | 2.44E-05 | 1.14E-05 |
|  | K02664 | 8.71E-05 | 8.29E-05 | 8.86E-05 | 8.25E-05 | 9.58E-05 | 8.27E-05 | 8.15E-05 | 2.96E-05 | 5.97E-05 | 2.44E-05 |
|  | K02674 | 2.08E-04 | 2.40E-04 | 2.07E-04 | 1.97E-04 | 2.13E-04 | 2.21E-04 | 7.50E-05 | 1.27E-04 | 6.09E-05 | 1.33E-04 |
|  | K02655 | 3.67E-05 | 1.79E-05 | 2.71E-05 | 2.05E-05 | 3.25E-05 | 2.59E-05 | 7.11E-06 | 1.95E-05 | 3.64E-06 | 1.90E-05 |
|  | K02665 | 3.72E-05 | 5.91E-05 | 5.08E-05 | 3.69E-05 | 4.56E-05 | 4.85E-05 | 5.39E-06 | 6.70E-06 | 2.70E-06 | 4.78E-06 |
|  | SUM | 2.49E-03 | 2.33E-03 | 2.53E-03 | 2.47E-03 | 2.41E-03 | 2.37E-03 | 1.83E-03 | 1.51E-03 | 1.95E-03 | 1.80E-03 |
| Cytochrome | K08738 | 3.57E-05 | 3.24E-05 | 3.49E-05 | 2.05E-05 | 3.91E-05 | 2.23E-05 | 1.63E-05 | 1.39E-05 | 1.69E-05 | 1.01E-05 |
|  | K02569 | 2.07E-05 | 6.41E-06 | 1.06E-05 | 5.21E-06 | 1.26E-05 | 9.74E-06 | 1.60E-05 | 9.65E-06 | 1.20E-05 | 8.38E-06 |
|  | K02300 | 1.07E-05 | 2.97E-06 | 7.11E-06 | 1.63E-06 | 5.57E-06 | 3.20E-06 | 5.77E-06 | 3.85E-06 | 4.76E-06 | 2.96E-06 |
|  | K02298 | 1.51E-05 | 6.54E-06 | 1.03E-05 | 2.97E-06 | 1.08E-05 | 5.32E-06 | 9.50E-06 | 8.51E-06 | 6.44E-06 | 4.91E-06 |
|  | K02297 | 1.23E-05 | 3.55E-06 | 6.22E-06 | 2.24E-06 | 8.13E-06 | 5.30E-06 | 7.30E-06 | 6.25E-06 | 4.15E-06 | 4.90E-06 |
|  | K02277 | 1.22E-05 | 8.10E-06 | 1.08E-05 | 7.97E-06 | 1.11E-05 | 1.47E-05 | 8.12E-06 | 6.19E-06 | 7.17E-06 | 6.90E-06 |
|  | K02276 | 3.91E-05 | 3.42E-05 | 3.64E-05 | 3.35E-05 | 3.45E-05 | 4.37E-05 | 2.22E-05 | 2.02E-05 | 2.28E-05 | 2.01E-05 |
|  | K02275 | 6.75E-05 | 6.43E-05 | 6.55E-05 | 6.56E-05 | 5.77E-05 | 7.28E-05 | 4.02E-05 | 3.68E-05 | 3.46E-05 | 3.34E-05 |
|  | K02274 | 8.18E-05 | 7.10E-05 | 7.16E-05 | 7.44E-05 | 6.75E-05 | 8.18E-05 | 4.66E-05 | 3.92E-05 | 4.66E-05 | 4.13E-05 |
|  | K02259 | 3.68E-05 | 3.68E-05 | 3.44E-05 | 3.65E-05 | 2.98E-05 | 4.14E-05 | 2.07E-05 | 1.71E-05 | 1.82E-05 | 1.77E-05 |
|  | K02200 | 7.18E-05 | 8.08E-05 | 7.95E-05 | 7.05E-05 | 7.83E-05 | 7.63E-05 | 8.85E-05 | 4.15E-05 | 9.25E-05 | 3.21E-05 |
|  | K02199 | 1.51E-05 | 1.68E-05 | 1.28E-05 | 1.46E-05 | 1.10E-05 | 0.000014 | 1.14E-05 | 1.10E-05 | 1.36E-05 | 9.91E-06 |
|  | K00413 | 2.04E-05 | 1.44E-05 | 2.35E-05 | 1.53E-05 | 1.24E-05 | 9.67E-06 | 5.98E-06 | 4.73E-06 | 5.90E-06 | 3.18E-06 |
|  | K00412 | 3.30E-05 | 2.89E-05 | 3.15E-05 | 2.31E-05 | 2.16E-05 | 2.09E-05 | 1.93E-05 | 1.82E-05 | 6.75E-05 | 1.70E-05 |
|  | K00411 | 2.02E-05 | 1.61E-05 | 2.06E-05 | 1.45E-05 | 1.25E-05 | 8.01E-06 | 6.65E-06 | 4.33E-06 | 4.58E-06 | 3.57E-06 |
|  | K00406 | 2.24E-05 | 1.78E-05 | 1.95E-05 | 1.38E-05 | 1.54E-05 | 1.39E-05 | 1.06E-05 | 8.78E-06 | 1.05E-05 | 8.80E-06 |
|  | K00405 | 2.03E-05 | 1.42E-05 | 2.16E-05 | 9.40E-06 | 2.31E-05 | 9.71E-06 | 8.44E-06 | 1.24E-05 | 7.93E-06 | 6.96E-06 |
|  | K00404 | 2.24E-05 | 1.86E-05 | 2.26E-05 | 8.89E-06 | 2.16E-05 | 1.33E-05 | 1.13E-05 | 1.05E-05 | 1.12E-05 | 7.65E-06 |
|  | SUM | 5.57E-04 | 4.74E-04 | 5.19E-04 | 4.21E-04 | 4.73E-04 | 4.66E-04 | 3.55E-04 | 2.73E-04 | 3.87E-04 | 2.40E-04 |
| Ubiquinone | K01075 | 2.62E-06 | 7.88E-07 | 4.15E-06 | 0.00E+00 | 8.30E-06 | 0.00E+00 | 0.00E+00 | 0.00E+00 | 0.00E+00 | 5.27E-07 |
|  | K03181 | 5.00E-06 | 5.16E-06 | 7.77E-06 | 2.86E-06 | 9.38E-06 | 3.10E-06 | 3.65E-06 | 2.49E-06 | 1.27E-06 | 3.19E-06 |
|  | K03179 | 3.19E-04 | 2.93E-04 | 3.05E-04 | 3.03E-04 | 2.99E-04 | 3.28E-04 | 3.18E-04 | 2.55E-04 | 2.74E-04 | 2.82E-04 |
|  | K03186 | 3.28E-04 | 3.11E-04 | 3.59E-04 | 3.44E-04 | 3.53E-04 | 3.47E-04 | 3.04E-04 | 2.72E-04 | 2.89E-04 | 2.75E-04 |
|  | K00568 | 3.50E-05 | 3.91E-05 | 4.19E-05 | 3.81E-05 | 3.94E-05 | 2.59E-05 | 2.64E-05 | 3.00E-05 | 3.17E-05 | 1.84E-05 |
|  | K03185 | 8.93E-06 | 6.94E-06 | 8.83E-06 | 4.39E-06 | 5.85E-06 | 3.28E-06 | 7.22E-06 | 7.09E-06 | 4.47E-06 | 3.35E-06 |
|  | K03183 | 4.59E-04 | 4.11E-04 | 4.37E-04 | 4.20E-04 | 4.55E-04 | 4.31E-04 | 2.67E-04 | 2.88E-04 | 3.05E-04 | 2.64E-04 |
|  | K06134 | 1.20E-05 | 9.48E-06 | 1.39E-05 | 7.15E-06 | 1.18E-05 | 4.81E-06 | 1.33E-05 | 1.25E-05 | 1.05E-05 | 8.86E-06 |
|  | K02361 | 5.32E-06 | 5.24E-06 | 5.75E-06 | 3.77E-06 | 3.46E-06 | 4.73E-06 | 1.16E-05 | 1.49E-05 | 1.64E-05 | 1.21E-05 |
|  | K02551 | 1.86E-05 | 2.32E-05 | 4.10E-05 | 2.22E-05 | 1.11E-05 | 2.16E-05 | 3.57E-05 | 4.58E-05 | 5.59E-05 | 4.28E-05 |
|  | K08680 | 2.64E-06 | 4.11E-06 | 0.00E+00 | 2.96E-06 | 0.00E+00 | 2.12E-06 | 1.18E-05 | 1.59E-05 | 2.55E-05 | 2.49E-05 |
|  | K02549 | 4.28E-05 | 3.83E-05 | 3.22E-05 | 3.77E-05 | 3.25E-05 | 4.13E-05 | 3.40E-05 | 4.24E-05 | 5.21E-05 | 4.34E-05 |
|  | K12073 | 1.28E-06 | 2.17E-06 | 0.00E+00 | 2.25E-06 | 0.00E+00 | 3.00E-06 | 8.05E-07 | 0.00E+00 | 0.00E+00 | 2.42E-06 |
|  | K19222 | 1.57E-06 | 1.98E-06 | 3.58E-06 | 3.16E-06 | 1.60E-06 | 1.83E-06 | 5.14E-07 | 0.00E+00 | 0.00E+00 | 0.00E+00 |
|  | K02548 | 5.92E-04 | 5.63E-04 | 6.25E-04 | 5.87E-04 | 6.59E-04 | 5.70E-04 | 6.11E-04 | 6.17E-04 | 7.08E-04 | 6.28E-04 |
|  | K11782 | 1.30E-04 | 1.18E-04 | 1.62E-04 | 1.42E-04 | 1.68E-04 | 1.48E-04 | 5.42E-05 | 6.16E-05 | 5.97E-05 | 4.54E-05 |
|  | K00355 | 6.42E-05 | 5.84E-05 | 9.82E-05 | 7.31E-05 | 9.82E-05 | 8.47E-05 | 6.13E-06 | 2.75E-05 | 3.19E-05 | 5.85E-06 |
|  | K03809 | 8.61E-05 | 8.40E-05 | 7.59E-05 | 8.85E-05 | 8.43E-05 | 8.05E-05 | 7.83E-05 | 5.00E-05 | 5.39E-05 | 6.25E-05 |
|  | K19267 | 6.79E-06 | 7.73E-06 | 6.37E-06 | 3.68E-06 | 5.16E-06 | 3.96E-06 | 1.31E-05 | 1.47E-05 | 1.34E-05 | 1.20E-05 |
|  | K00457 | 1.13E-05 | 4.03E-06 | 1.08E-05 | 3.88E-06 | 1.56E-05 | 6.37E-06 | 2.15E-06 | 0.00E+00 | 5.43E-07 | 2.18E-06 |
|  | SUM | 2.13E-03 | 1.99E-03 | 2.24E-03 | 2.09E-03 | 2.26E-03 | 2.11E-03 | 1.80E-03 | 1.76E-03 | 1.93E-03 | 1.74E-03 |

# Table S7 The abundance and description energy metabolism related genes in control and QS enhancement systems.

| K_ID | Description | Propanol-feed | | | | | | Butanol-feed | | | |
| --- | --- | --- | --- | --- | --- | --- | --- | --- | --- | --- | --- |
|  |  | Stage VII | | Stage VIII | | Stage IV | | Stage VIII | | Stage IV | |
|  |  | QS | Con | QS | Con | QS | Con | QS | Con | QS | Con |
| K00330 | NADH-quinone oxidoreductase subunit A | 3.94E-04 | 3.43E-04 | 3.94E-04 | 3.48E-04 | 4.04E-04 | 3.51E-04 | 4.03E-04 | 3.39E-04 | 4.10E-04 | 3.82E-04 |
| K00331 | NADH-quinone oxidoreductase subunit B | 3.68E-04 | 3.50E-04 | 3.61E-04 | 3.69E-04 | 3.92E-04 | 3.46E-04 | 3.91E-04 | 3.47E-04 | 3.92E-04 | 3.96E-04 |
| K00332 | NADH-quinone oxidoreductase subunit C | 2.46E-04 | 2.15E-04 | 2.60E-04 | 2.36E-04 | 2.73E-04 | 2.57E-04 | 2.12E-04 | 1.57E-04 | 1.89E-04 | 1.78E-04 |
| K00333 | NADH-quinone oxidoreductase subunit D | 2.59E-04 | 2.19E-04 | 2.67E-04 | 2.25E-04 | 2.64E-04 | 2.62E-04 | 2.48E-04 | 2.54E-04 | 2.24E-04 | 3.15E-04 |
| K13378 | NADH-quinone oxidoreductase subunit C/D | 1.43E-04 | 1.21E-04 | 1.29E-04 | 1.22E-04 | 1.21E-04 | 1.25E-04 | 6.22E-05 | 4.09E-05 | 5.83E-05 | 3.08E-05 |
| K00334 | NADH-quinone oxidoreductase subunit E | 1.48E-03 | 1.47E-03 | 1.64E-03 | 1.54E-03 | 1.65E-03 | 1.51E-03 | 1.29E-03 | 1.10E-03 | 1.24E-03 | 1.16E-03 |
| K00335 | NADH-quinone oxidoreductase subunit F | 1.78E-03 | 1.76E-03 | 1.94E-03 | 1.80E-03 | 2.01E-03 | 1.84E-03 | 1.52E-03 | 1.38E-03 | 1.45E-03 | 1.50E-03 |
| K00336 | NADH-quinone oxidoreductase subunit G | 5.69E-04 | 5.87E-04 | 5.63E-04 | 5.58E-04 | 5.62E-04 | 5.44E-04 | 4.84E-04 | 4.63E-04 | 4.54E-04 | 5.42E-04 |
| K00337 | NADH-quinone oxidoreductase subunit H | 5.02E-04 | 4.55E-04 | 4.93E-04 | 4.70E-04 | 4.88E-04 | 4.73E-04 | 5.34E-04 | 4.87E-04 | 5.63E-04 | 5.43E-04 |
| K00338 | NADH-quinone oxidoreductase subunit I | 3.89E-04 | 2.95E-04 | 3.72E-04 | 3.43E-04 | 3.67E-04 | 3.34E-04 | 4.11E-04 | 3.46E-04 | 3.78E-04 | 3.99E-04 |
| K00339 | NADH-quinone oxidoreductase subunit J | 4.70E-04 | 3.65E-04 | 4.26E-04 | 4.02E-04 | 4.47E-04 | 4.30E-04 | 5.22E-04 | 4.58E-04 | 5.11E-04 | 5.01E-04 |
| K00340 | NADH-quinone oxidoreductase subunit K | 4.08E-04 | 3.39E-04 | 3.83E-04 | 3.57E-04 | 3.87E-04 | 3.59E-04 | 4.31E-04 | 3.28E-04 | 4.04E-04 | 3.72E-04 |
| K00341 | NADH-quinone oxidoreductase subunit L | 5.23E-04 | 4.50E-04 | 4.97E-04 | 4.41E-04 | 4.97E-04 | 4.92E-04 | 5.34E-04 | 4.34E-04 | 4.96E-04 | 4.90E-04 |
| K00342 | NADH-quinone oxidoreductase subunit M | 5.88E-04 | 5.09E-04 | 5.62E-04 | 5.20E-04 | 5.75E-04 | 5.23E-04 | 5.70E-04 | 4.50E-04 | 5.54E-04 | 4.60E-04 |
| K00343 | NADH-quinone oxidoreductase subunit N | 4.77E-04 | 4.35E-04 | 4.51E-04 | 4.23E-04 | 4.34E-04 | 4.29E-04 | 4.17E-04 | 3.77E-04 | 4.03E-04 | 4.45E-04 |
| K00331 | NADH-quinone oxidoreductase subunit B | 3.68E-04 | 3.50E-04 | 3.61E-04 | 3.69E-04 | 3.92E-04 | 3.46E-04 | 3.91E-04 | 3.47E-04 | 3.92E-04 | 3.96E-04 |
| K13380 | NADH-quinone oxidoreductase subunit B/C/D | 2.59E-05 | 1.69E-05 | 2.68E-05 | 1.93E-05 | 3.52E-05 | 2.40E-05 | 1.27E-05 | 4.84E-06 | 9.02E-06 | 4.34E-06 |
| SUM |  | 8.98E-03 | 8.28E-03 | 9.12E-03 | 8.54E-03 | 9.29E-03 | 8.65E-03 | 8.44E-03 | 7.32E-03 | 8.13E-03 | 8.12E-03 |
| K00240 | succinate dehydrogenase / fumarate reductase, iron-sulfur subunit | 2.55E-04 | 2.58E-04 | 2.71E-04 | 2.77E-04 | 2.86E-04 | 2.82E-04 | 2.89E-04 | 1.82E-04 | 3.10E-04 | 1.82E-04 |
| K00239 | succinate dehydrogenase / fumarate reductase, flavoprotein subunit | 2.66E-04 | 2.48E-04 | 2.37E-04 | 2.43E-04 | 2.40E-04 | 2.60E-04 | 3.02E-04 | 2.14E-04 | 2.75E-04 | 1.93E-04 |
| K00242 | succinate dehydrogenase / fumarate reductase, membrane anchor subunit | 1.57E-04 | 9.95E-05 | 1.20E-04 | 1.03E-04 | 1.13E-04 | 1.03E-04 | 9.56E-05 | 4.64E-05 | 1.14E-04 | 3.31E-05 |
| K00241 | succinate dehydrogenase / fumarate reductase, cytochrome b subunit | 2.35E-04 | 1.91E-04 | 1.95E-04 | 1.95E-04 | 1.98E-04 | 2.10E-04 | 1.61E-04 | 1.03E-04 | 1.91E-04 | 7.42E-05 |
| K00244 | fumarate reductase flavoprotein subunit | 9.24E-05 | 6.94E-05 | 8.07E-05 | 6.70E-05 | 8.87E-05 | 7.01E-05 | 9.25E-05 | 2.51E-04 | 6.74E-05 | 1.81E-04 |
| K00245 | fumarate reductase iron-sulfur subunit | 5.51E-05 | 3.98E-05 | 5.75E-05 | 4.42E-05 | 7.01E-05 | 4.69E-05 | 2.01E-05 | 1.05E-05 | 1.34E-05 | 1.15E-05 |
| K00246 | fumarate reductase subunit C | 4.36E-05 | 3.39E-05 | 4.79E-05 | 3.95E-05 | 5.59E-05 | 3.99E-05 | 1.50E-05 | 7.90E-06 | 1.14E-05 | 9.31E-06 |
| SUM |  | 1.10E-03 | 9.40E-04 | 1.01E-03 | 9.69E-04 | 1.05E-03 | 1.01E-03 | 9.75E-04 | 8.15E-04 | 9.83E-04 | 6.84E-04 |
| K00425 | cytochrome d ubiquinol oxidase subunit I | 2.60E-04 | 2.16E-04 | 2.52E-04 | 2.35E-04 | 2.72E-04 | 2.69E-04 | 1.90E-04 | 1.16E-04 | 1.75E-04 | 9.74E-05 |
| K00426 | cytochrome d ubiquinol oxidase subunit II | 2.43E-04 | 2.17E-04 | 2.38E-04 | 2.44E-04 | 2.48E-04 | 2.65E-04 | 2.02E-04 | 1.63E-04 | 1.87E-04 | 1.51E-04 |
| SUM |  | 5.03E-04 | 4.33E-04 | 4.90E-04 | 4.80E-04 | 5.20E-04 | 5.34E-04 | 3.93E-04 | 2.79E-04 | 3.63E-04 | 2.48E-04 |
| K02275 | cytochrome c oxidase subunit II | 6.75E-05 | 6.43E-05 | 6.55E-05 | 6.56E-05 | 5.77E-05 | 7.28E-05 | 4.02E-05 | 3.68E-05 | 3.46E-05 | 3.34E-05 |
| K02274 | cytochrome c oxidase subunit I | 8.18E-05 | 7.10E-05 | 7.16E-05 | 7.44E-05 | 6.75E-05 | 8.18E-05 | 4.66E-05 | 3.92E-05 | 4.66E-05 | 4.13E-05 |
| K02276 | cytochrome c oxidase subunit III | 3.91E-05 | 3.42E-05 | 3.64E-05 | 3.35E-05 | 3.45E-05 | 4.37E-05 | 2.22E-05 | 2.02E-05 | 2.28E-05 | 2.01E-05 |
| K02277 | cytochrome c oxidase subunit IV | 1.22E-05 | 8.10E-06 | 1.08E-05 | 7.97E-06 | 1.11E-05 | 1.47E-05 | 8.12E-06 | 6.19E-06 | 7.17E-06 | 6.90E-06 |
| K00404 | cytochrome c oxidase cbb3-type subunit I | 2.24E-05 | 1.86E-05 | 2.26E-05 | 8.89E-06 | 2.16E-05 | 1.33E-05 | 1.13E-05 | 1.05E-05 | 1.12E-05 | 7.65E-06 |
| K00405 | cytochrome c oxidase cbb3-type subunit II | 2.03E-05 | 1.42E-05 | 2.16E-05 | 9.40E-06 | 2.31E-05 | 9.71E-06 | 8.44E-06 | 1.24E-05 | 7.93E-06 | 6.96E-06 |
| K15862 | cytochrome c oxidase cbb3-type subunit I/II | 6.00E-06 | 2.58E-06 | 9.89E-07 | 2.05E-06 | 1.70E-06 | 4.55E-06 | 5.04E-06 | 5.94E-07 | 6.94E-06 | 5.15E-07 |
| K00407 | cytochrome c oxidase cbb3-type subunit IV | 1.26E-05 | 9.00E-06 | 1.14E-05 | 5.52E-06 | 7.90E-06 | 1.17E-06 | 4.12E-06 | 4.30E-06 | 2.49E-06 | 3.73E-06 |
| K00406 | cytochrome c oxidase cbb3-type subunit III | 2.24E-05 | 1.78E-05 | 1.95E-05 | 1.38E-05 | 1.54E-05 | 1.39E-05 | 1.06E-05 | 8.78E-06 | 1.05E-05 | 8.80E-06 |
| SUM |  | 2.84E-04 | 2.40E-04 | 2.60E-04 | 2.21E-04 | 2.41E-04 | 2.56E-04 | 1.57E-04 | 1.39E-04 | 1.50E-04 | 1.29E-04 |

# Table S8 The abundance and description related genes of electron-accepting in methanogens for control and QS enhancement systems.

# Table S9 The reaction with redox intermediates participated of each process.

|  | Reaction |
| --- | --- |
| 1 | Propane-1-ol + NAD+ <=> Propanal + NADH + H+ |
| 2 | Propanal + NAD+ + CoA <=> Propanoyl-CoA + NADH + H+ |
| 4 | Butanal + NADH + H+ <=> 1-Butanol + NAD+ |
| 5 | Butanal + CoA + NAD+ <=> Butanoyl-CoA + NADH + H+ |
| 9 | Quinone + Succinate <=> Hydroquinone + Fumarate |
| 11 | (S)-Malate + NAD+ <=> Pyruvate + CO2 + NADH + H+ |
| 12 | 2 Reduced ferredoxin + Acetyl-CoA + CO2 + 2 H+ <=> 2 Oxidized ferredoxin + Pyruvate + CoA |
| 13 | Electron-transferring flavoprotein + Propanoyl-CoA <=> Reduced electron-transferring flavoprotein + Propenoyl-CoA |
| 15 | 3-Oxopropanoate + CoA + NADP+ <=> Malonyl-CoA + NADPH + H+ |
| 17 | 3-Oxopropanoate + CoA + NAD+ <=> Acetyl-CoA + CO2 + NADH + H+ |
| 18 | Butanoyl-CoA + NAD+ <=> Crotonoyl-CoA + NADH + H+ |
| 20 | (S)-3-Hydroxybutanoyl-CoA + NADP+ <=> Acetoacetyl-CoA + NADPH + H+ |
| 26 | Coenzyme B + Coenzyme M + 2 Oxidized ferredoxin <=> Coenzyme M 7-mercaptoheptanoylthreonine-phosphate heterodisulfide + 2 Reduced ferredoxin + 2 H+ |
| 29 | 5-Methyltetrahydrofolate + NAD+ <=> 5,10-Methylenetetrahydrofolate + NADH + H+ |
| 30 | 5,10-Methylenetetrahydrofolate + NADP+ <=> 5,10-Methenyltetrahydrofolate + NADPH |
| 33 | Formylmethanofuran + 2 Oxidized ferredoxin + H2O <=> Methanofuran + 2 Reduced ferredoxin + CO2 + 2 H+ |
| 35 | 5,10-Methylenetetrahydromethanopterin + Coenzyme F420 + H+ <=> 5,10-Methenyltetrahydromethanopterin + Reduced coenzyme F420 |
| 36 | 5,10-Methylenetetrahydromethanopterin + Reduced coenzyme F420 <=> 5-Methyl-5,6,7,8-tetrahydromethanopterin + Coenzyme F420 |

# Table S10 Pearson correlation coefficient among metabolic capability community assembly, interspecies electron transfer, and community composition

| Interaction | AD | BES | DN |
| --- | --- | --- | --- |
| Electron transfer-Metabolic capability | 0.8 | 0.38 | 0.6 |
| Electron transfer-Community assembly | 0.64 | 0.26 | 0.44 |
| Metabolic capability-Community assembly | 0.28 | 0.6 | 0.57 |
| Community assembly-Microbic succession | 0.64 | 0.9 | 0.63 |

Note: AD means anaerobic digestion; BES means bioelectrochemical systems; DN means: anammox reactors


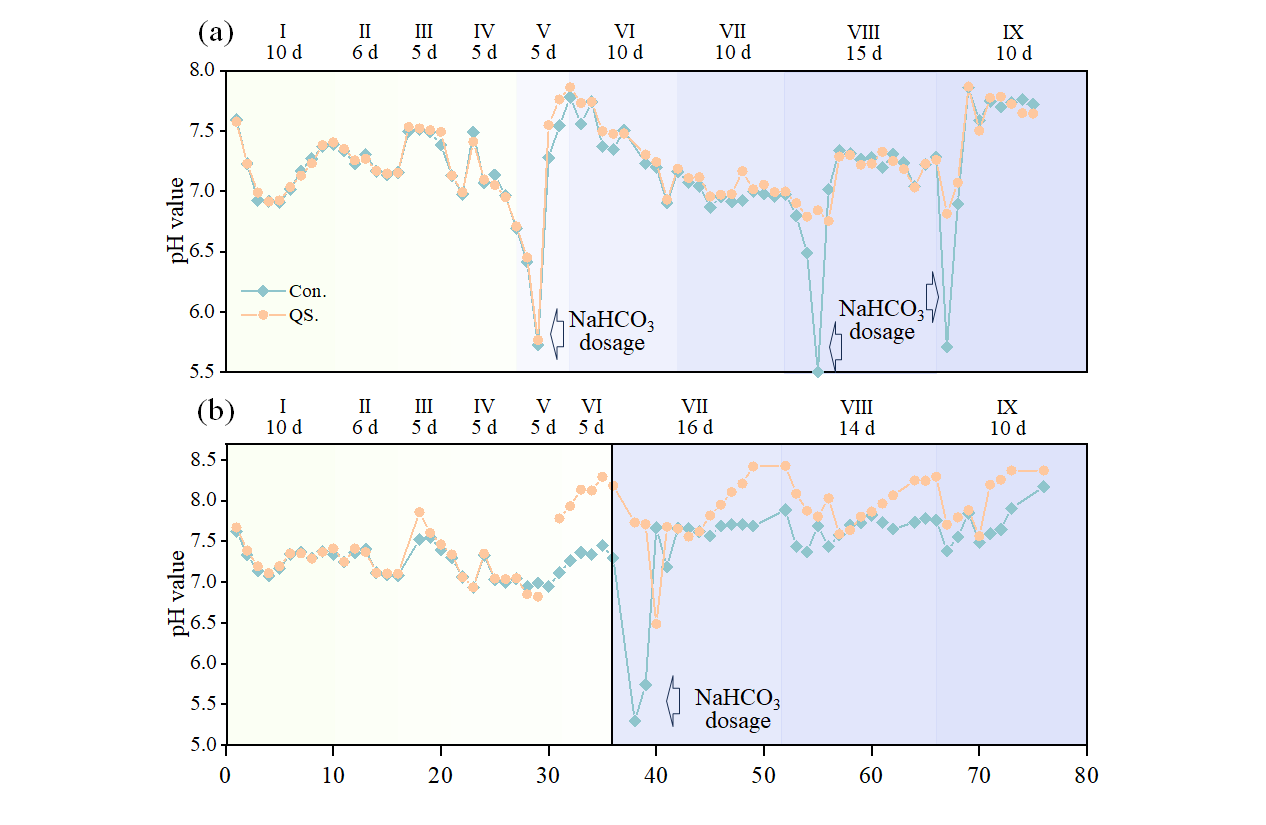


# Fig. S1 The variation of pH value in propanol-feed systems (a) and butanol-feed systems (b).


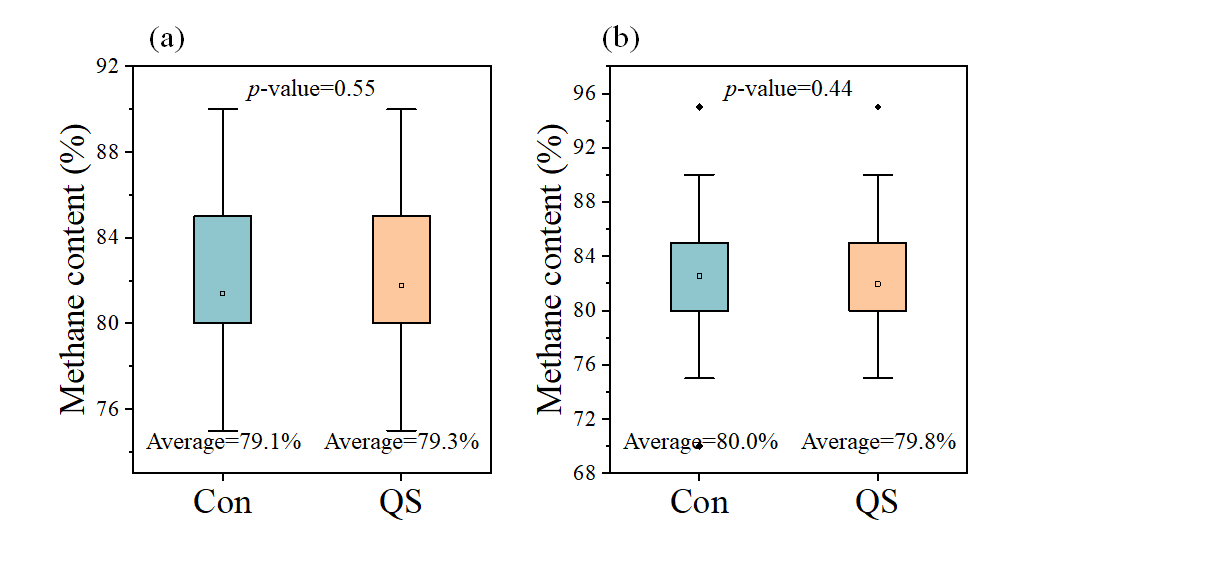


# Fig. S2 The variation of methane content in propanol- and butanol-feed systems.


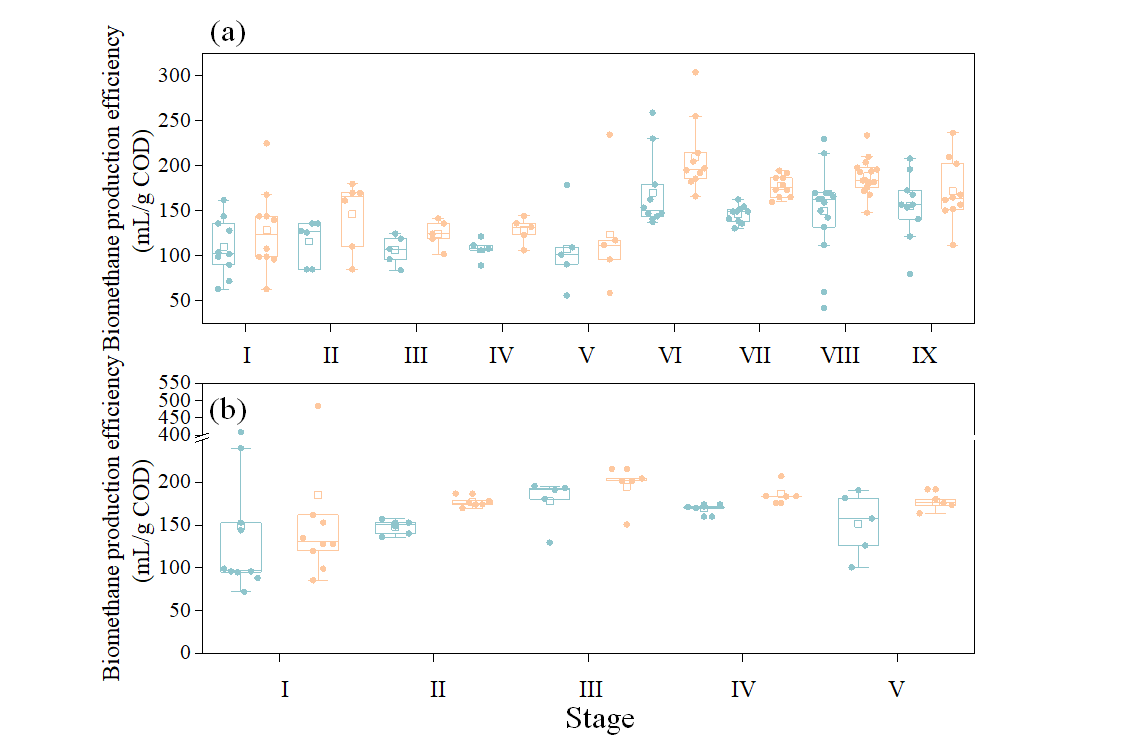


# Fig. S3 The boxplots of biomethane production efficiency in propanol-feed systems (a) and butanol-feed systems (b).


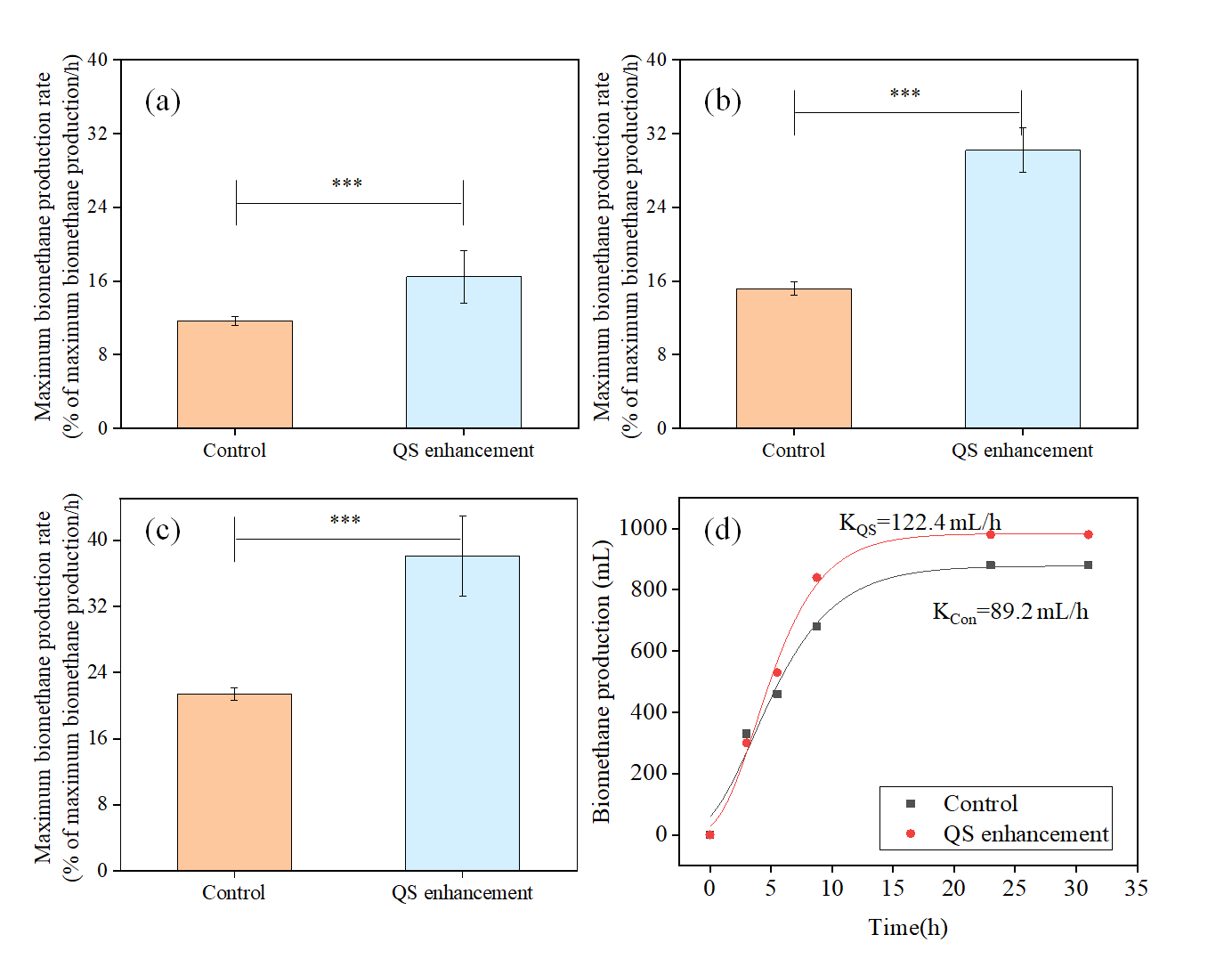


# Fig. S4 The maximum biomethane production rate at Stage VII (a), Stage VIII (b), and Stage IX (c) in butanol-feed systems; and the maximum biomethane production rate on Day 67 in propanol-systems. Note: The *** means the p-value<0.01.


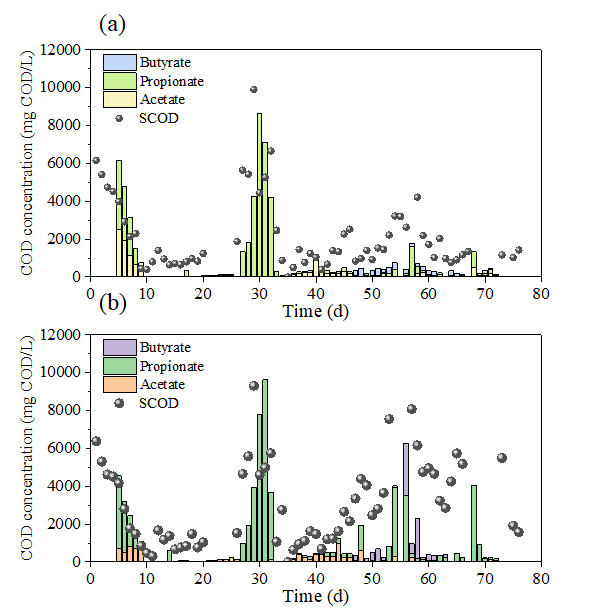


# Fig. S5 The SCOD, acetate, propionate and butyrate concentration changes in effluent of QS enhancement (a) and Control (b) systems during operation time for propanol-feed systems.


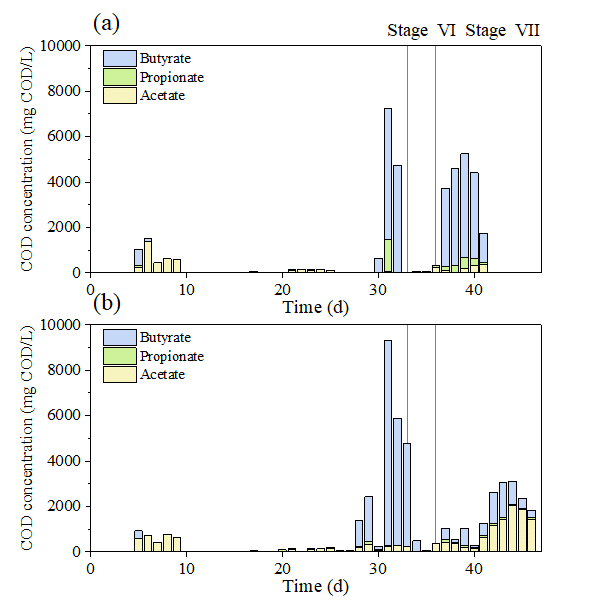


# Fig. S6 The acetate, propionate and butyrate concentration changes in effluent of QS enhancement (a) and Control (b) systems during operation time for butanol-feed systems.


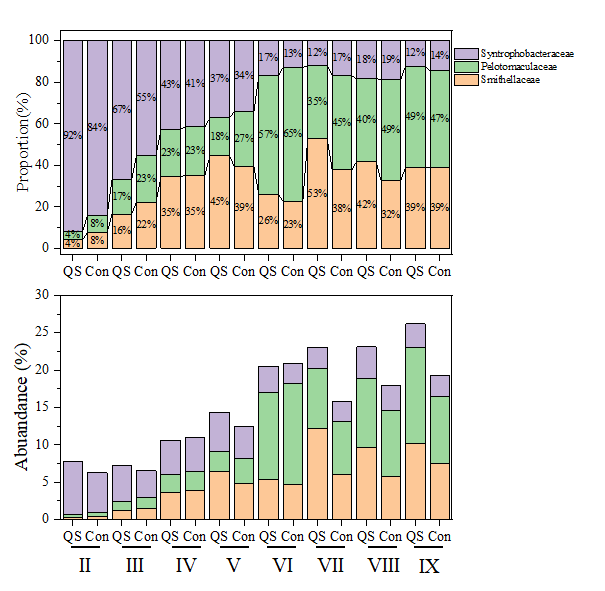


# Fig. S7 The abundance (a) and proportion of *Pelotomaculaceae*, *Syntrophobacteraceae*, and *Smithellaceae* in QS enhancement system and Control for propanol-feed systems.


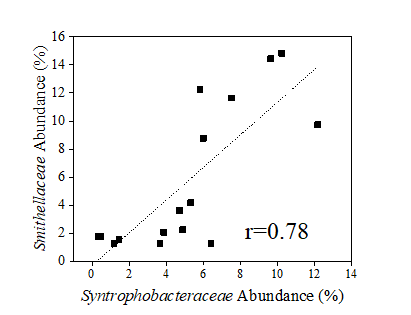


# Fig. S8 The correlation analysis of *Smithellaceae* and *Syntrophobacteraceae* for propanol-feed systems.


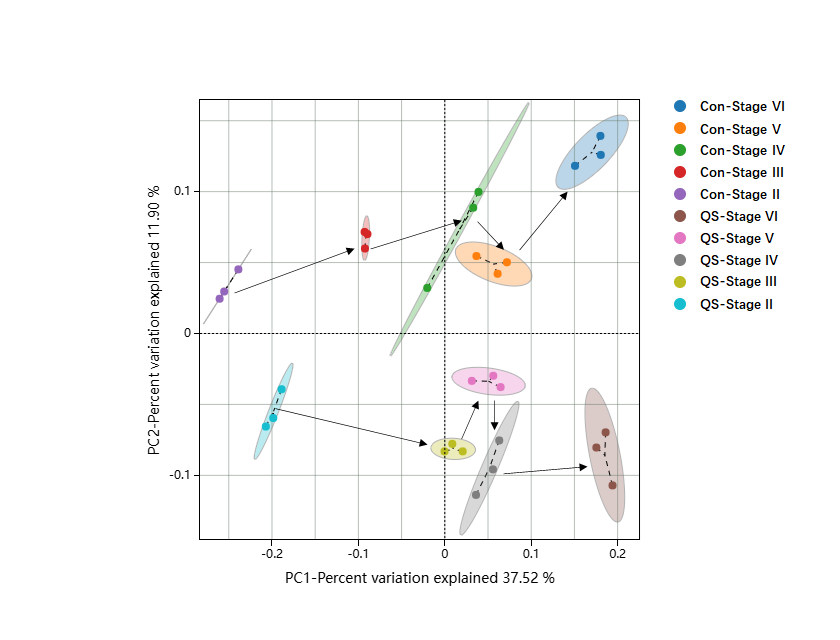


# Fig. S9 The PCA analysis of microbiota for butanol-feed systems.


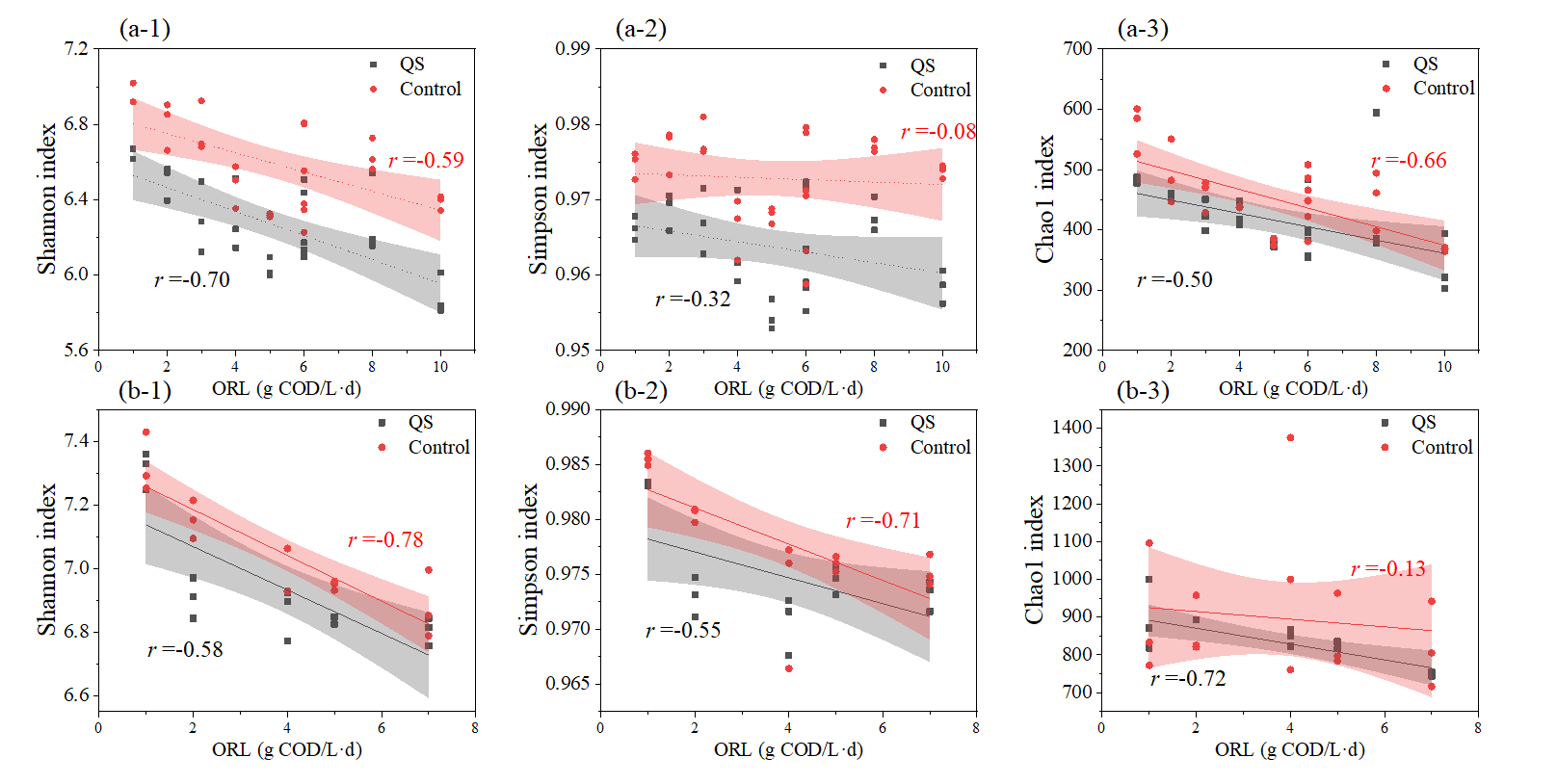


# Fig. S10 The correlations between α-diversity and ORL within propanol-feed systems (a) and butanol-feed systems (b).


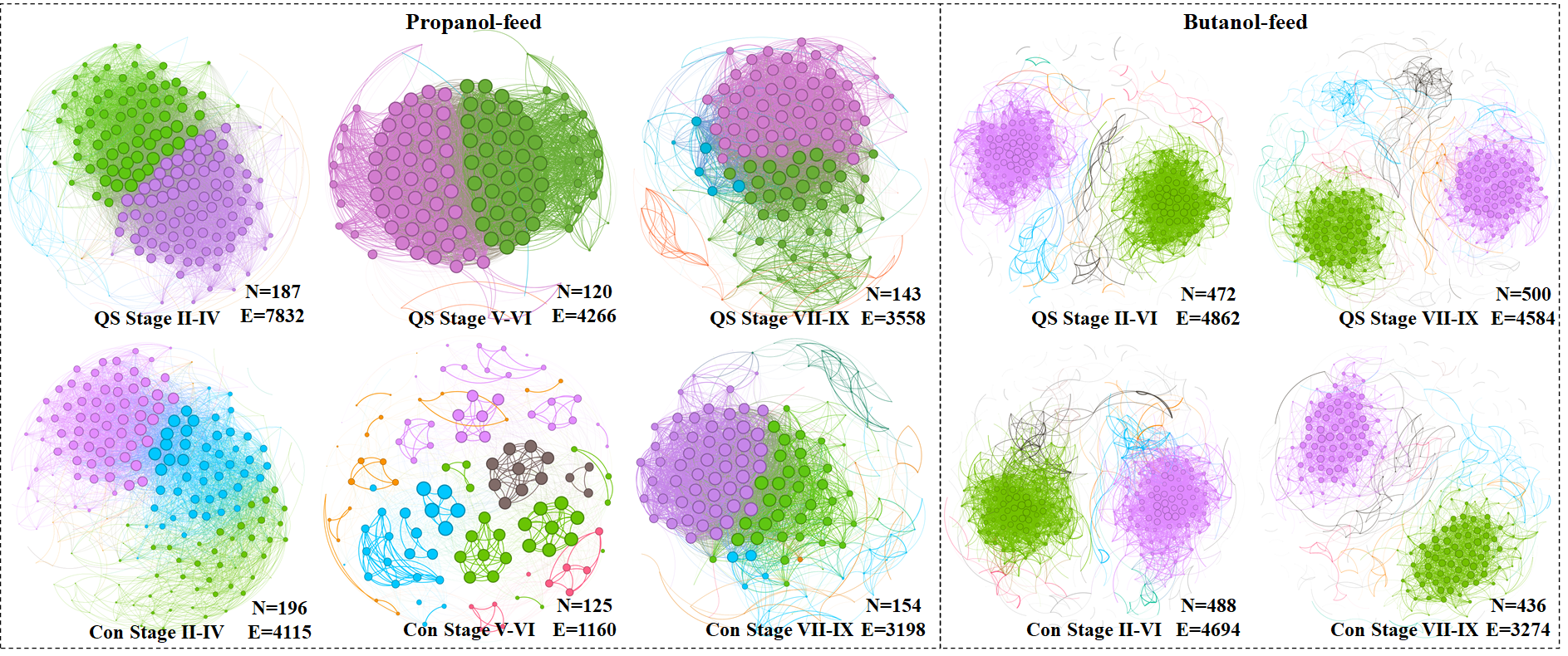


# Fig. S11 Microbial co-occurrence networks in QS enhancement and Con groups. n: number of nodes; e: number of edges. Nodes in each network are colored by modularity.


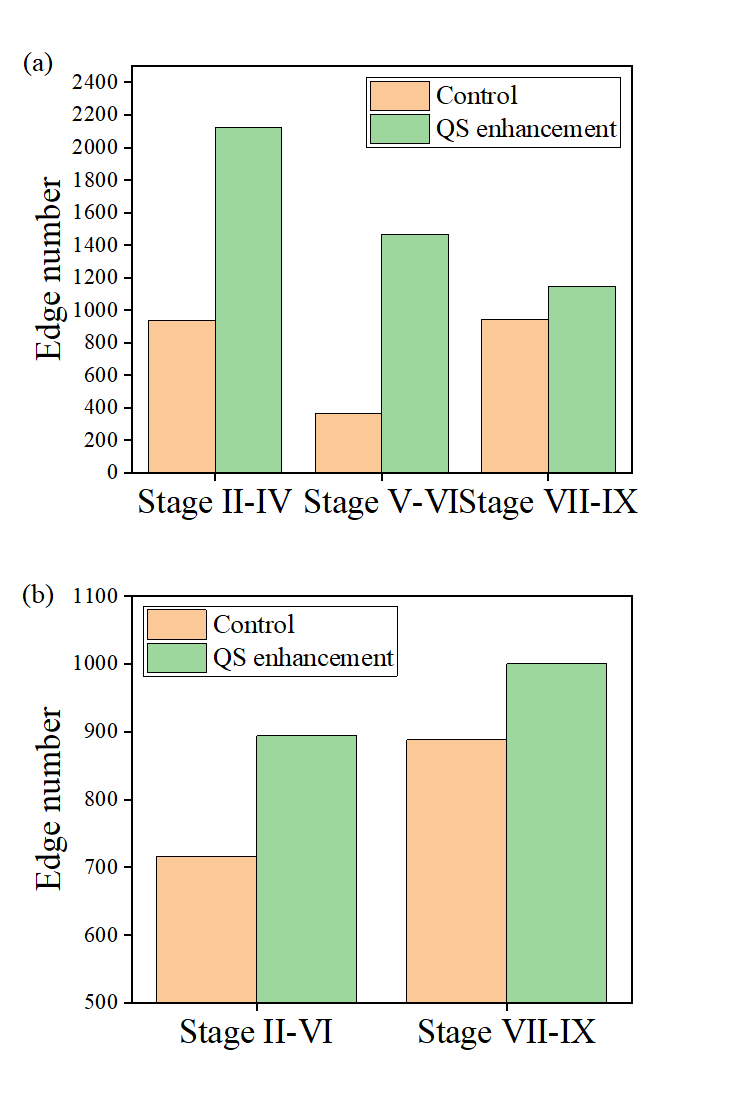


# Fig. S12 The number of edges connected to key microbial nodes in the network within propanol-feed systems (a) and butanol-feed systems (b).


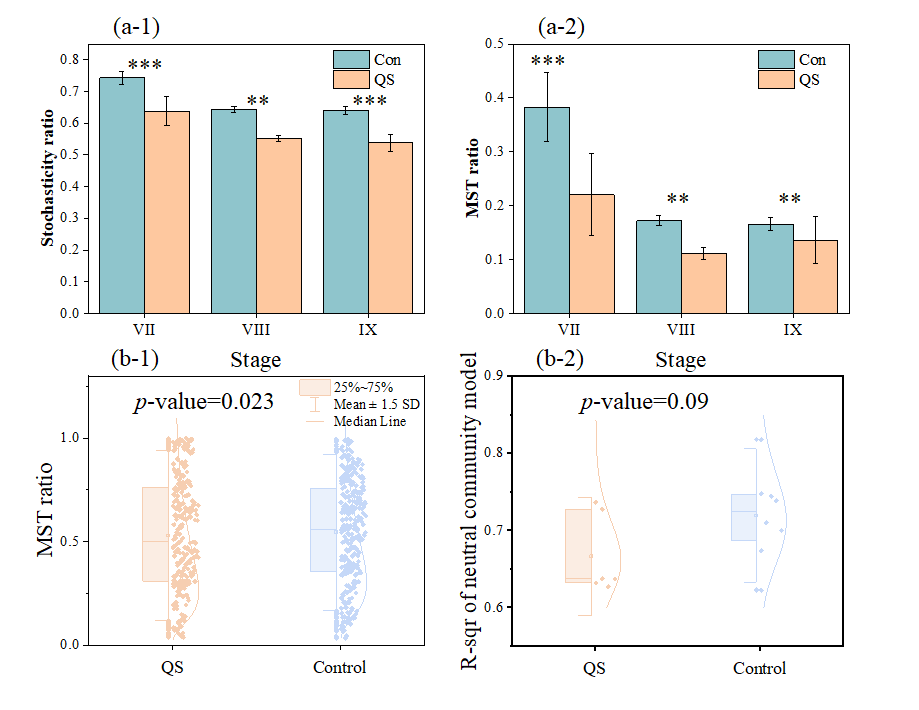


# Fig. S13 The stochasticity ratio (a-1) and MST ratio (a-2) values of butanol-feed systems; the MST ratio (b-1), R_2_ of neutral community model (b-2). The ** means the *p*-value<0.05; The *** means the *p*-value<0.01.


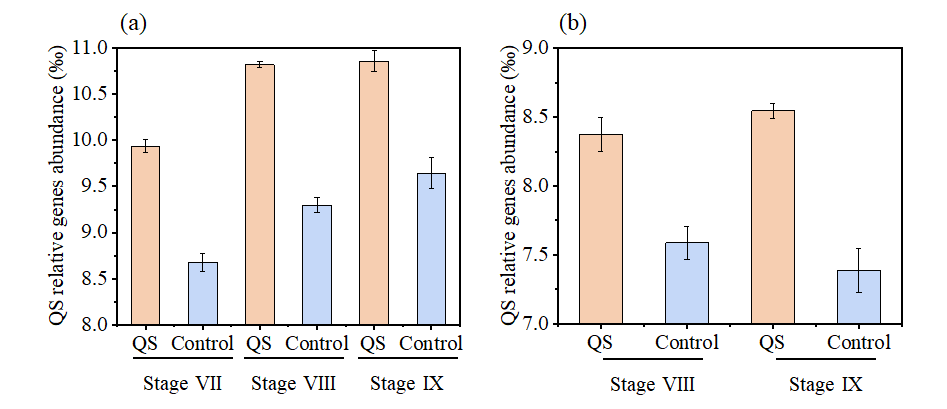


# Fig. S14 The gene abundance for quorum sensing (QS) in control and quorum sensing systems for propanol-feed systems (a) and butanol-feed systems (b)..


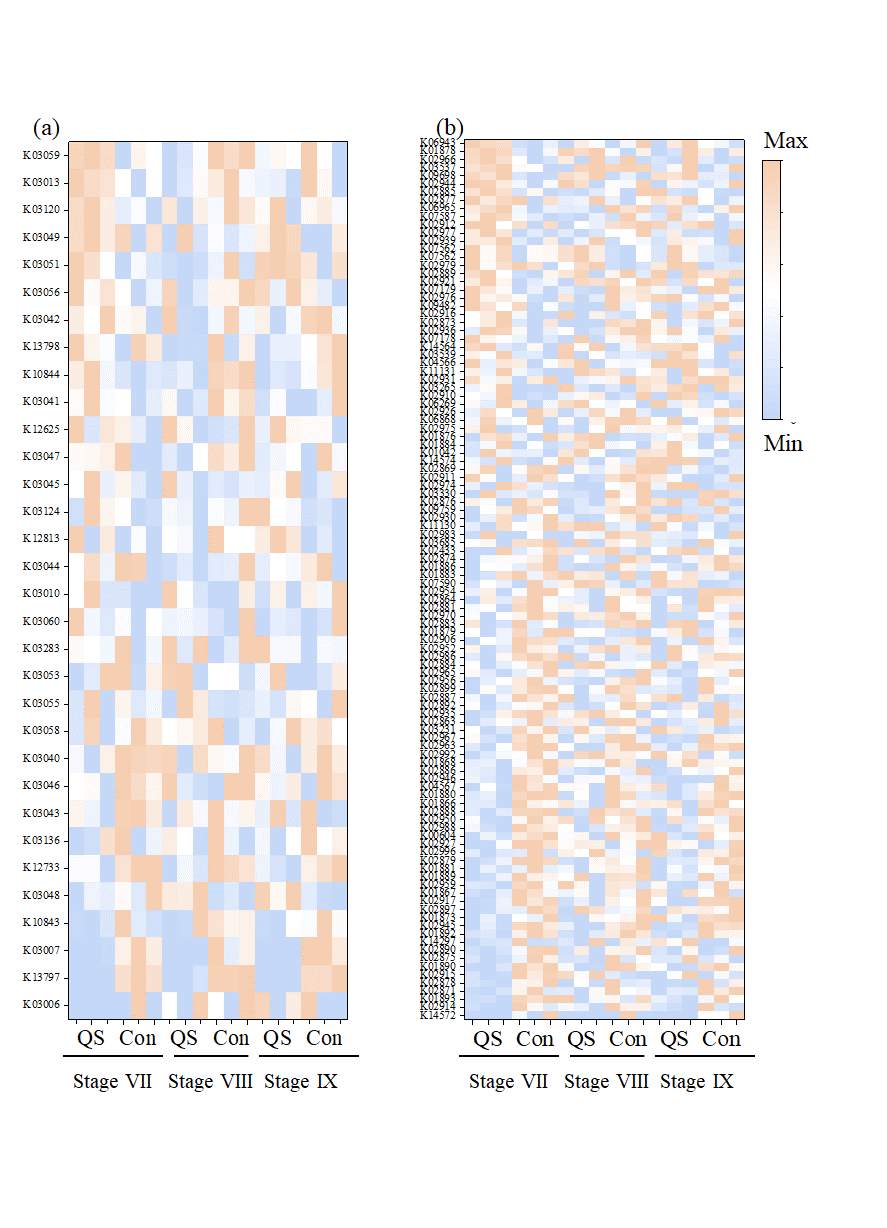


# Fig. S15 The gene expression of transcription (a) and translation (b) process in control and quorum sensing systems based on KEGG classifications for propanol-feed systems.


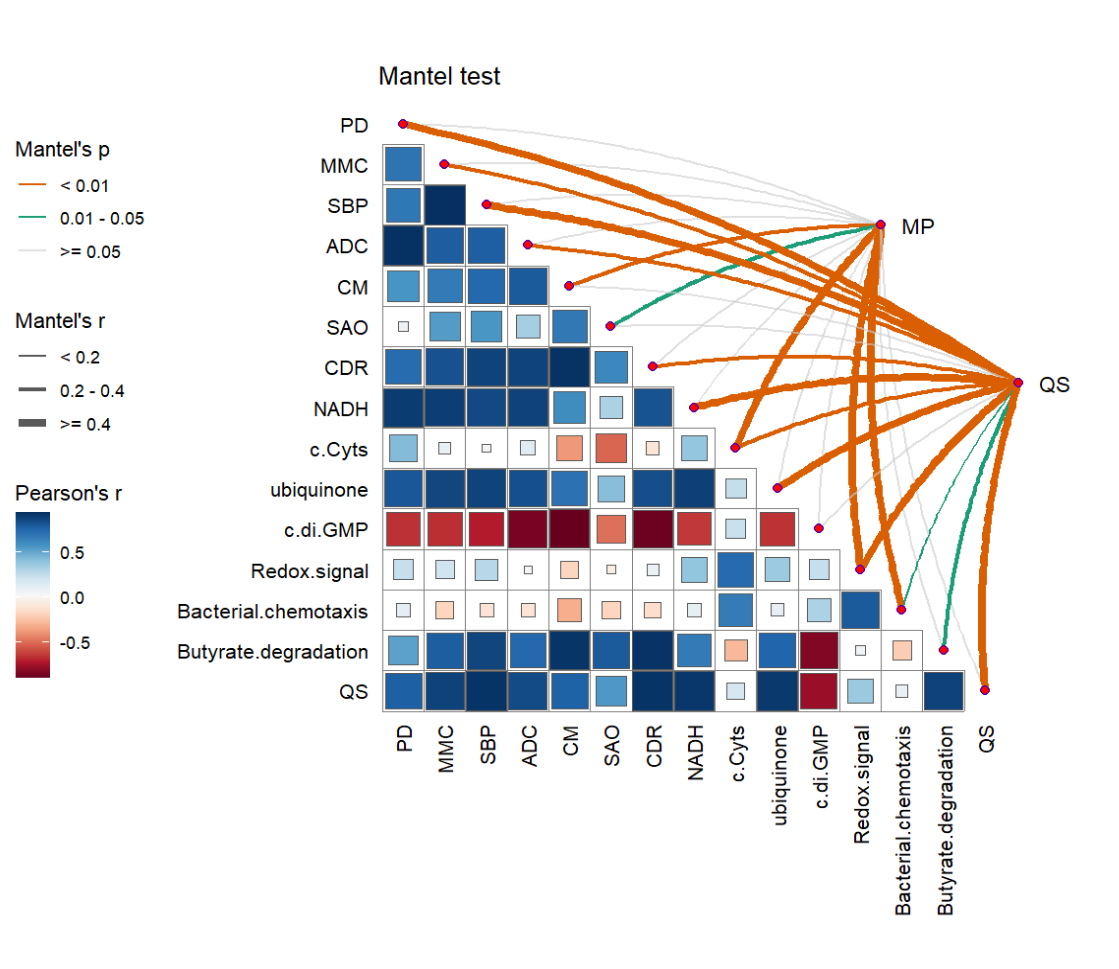


# Fig. S16 The Mantel test analyses of candidate key genes.


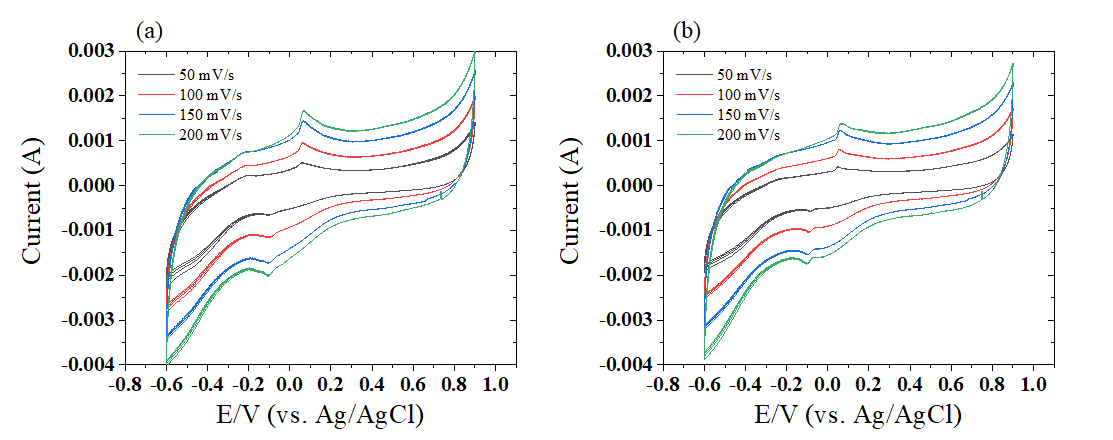


# Fig. S17 The cyclic voltammograms curves at different scan rates of QS enhancement system (a) and Control (b) for propanol-feed systems.


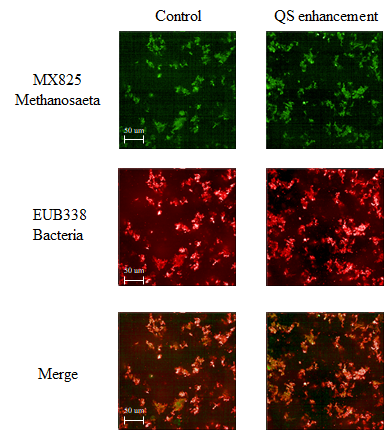


# Fig. S18 Fluorescence in situ hybridization images of sludge. The optimal images of sludge with *Bacteria* and *Methanosaeta* in control and quorum sensing systems at Stage VII for propanol-feed systems.


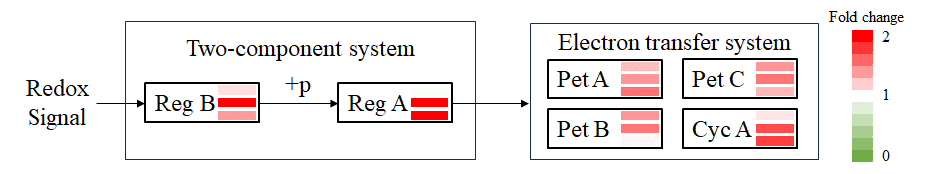


# Fig. S19 The metagenomic genes abundance of electron transfer system for propanol-feed systems. The shade of the color represents fold change values from these genes, and the upper block, average block, and lower block represents values in Stage VII, VIII, and IX, respectively.


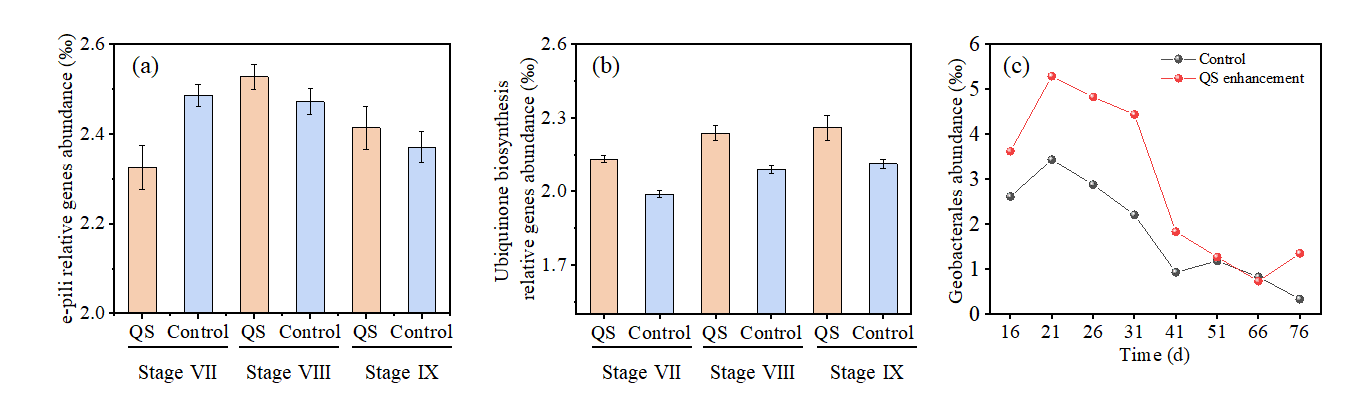


# Fig. S20 The Geobacterales abundance changes for propanol-feed systems


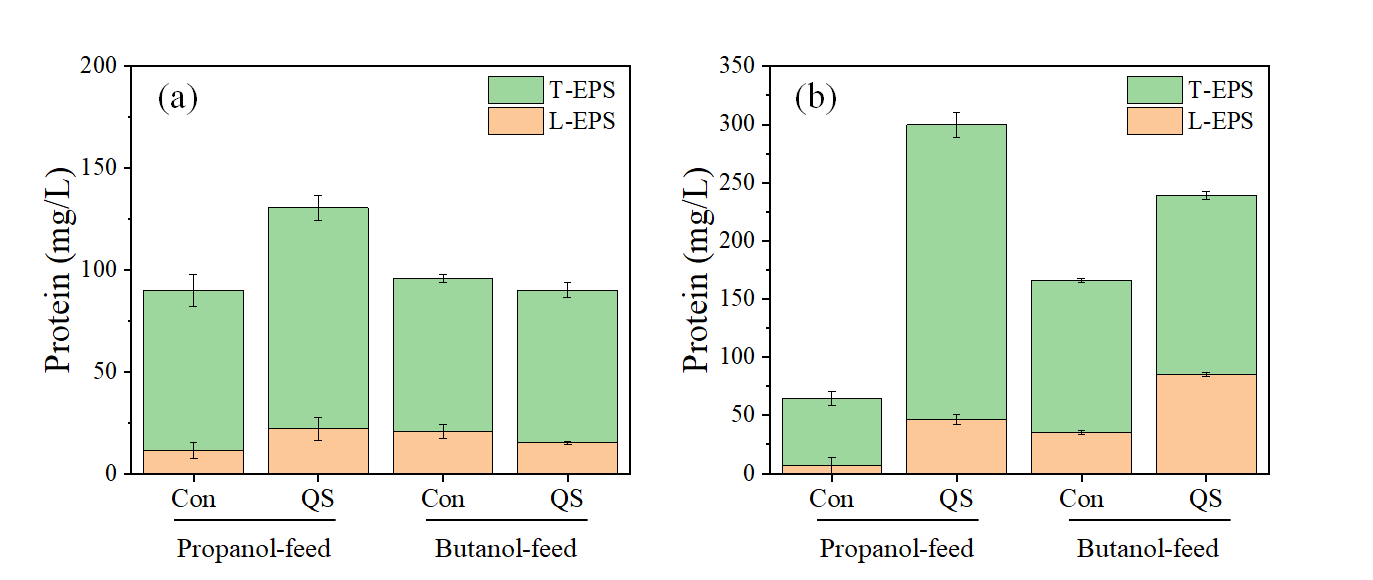


# Fig. S21 The protein characteristics of L-EPS and T-EPS variation in control and quorum sensing systems at Stage II (a) and Stage V (b) for propanol-feed systems and butanol-feed systems.


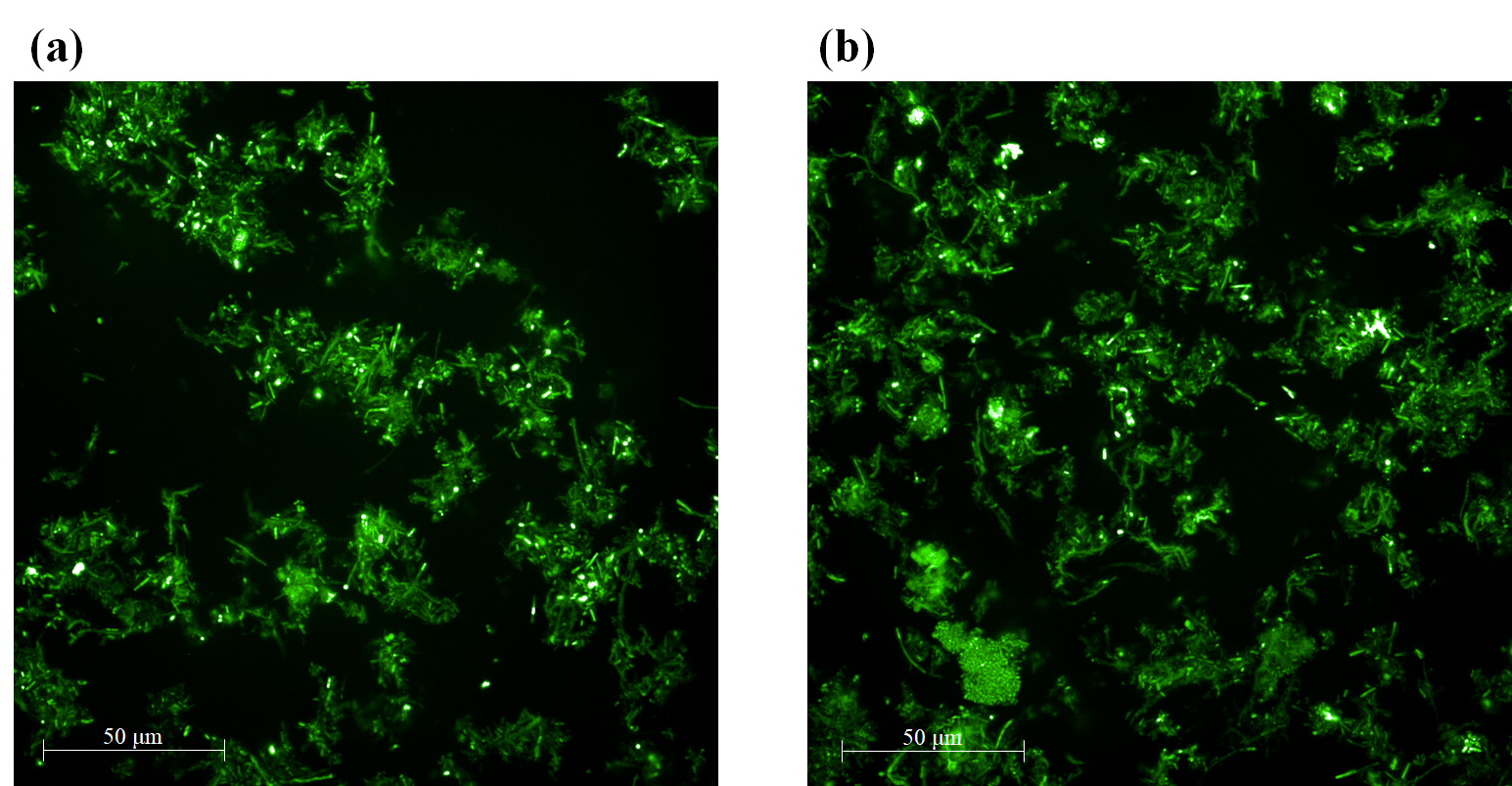


# Fig. S22 The CLSM images of cryosectioned sludge after FITC staining of Con (a) and QS enhancement (b) for propanol-feed systems.


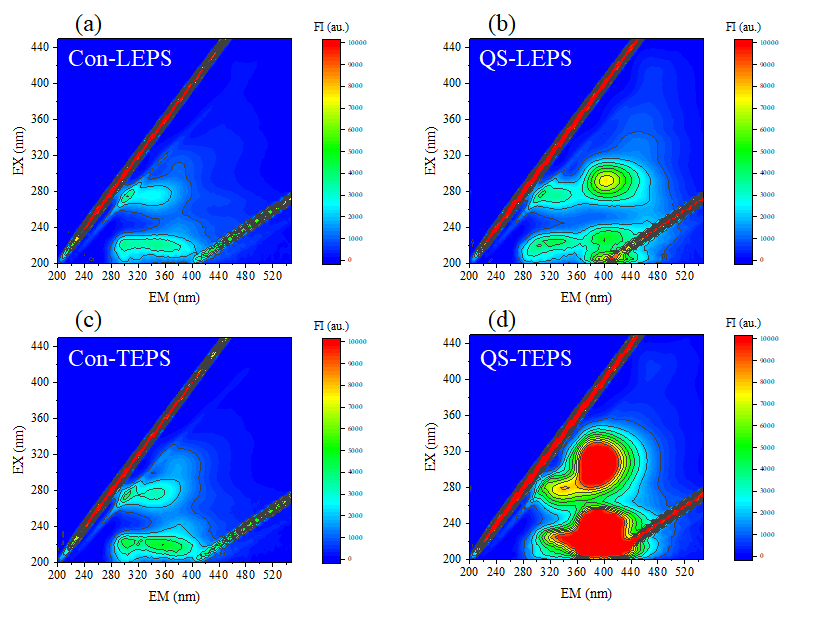


# Fig. S23 The fluorescence excitation–emission matrix spectra of EPS samples at Stage V for propanol-feed systems.


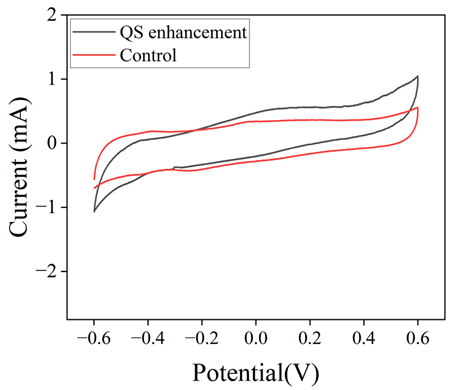


# Fig. S24 The cyclic voltammograms curves of EPS in QS enhancement system and Control at Stage V for propanol-feed systems.


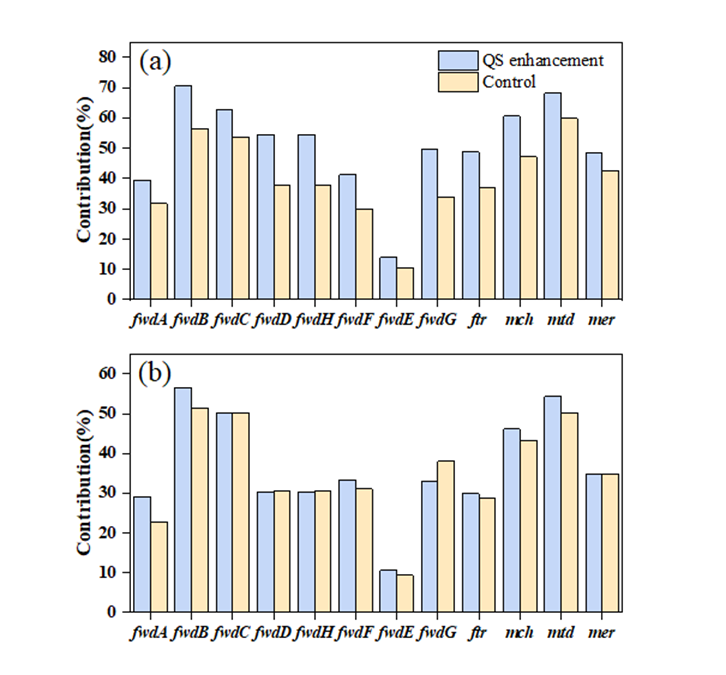


# Fig. S25 The genes expression proportion of *Methanothrix* in sludge about genes encoding the enzymes involved in carbon dioxide reduction. (Note: *fwd*A means formylmethanofuran dehydrogenase subunit A; *fwd*B means formylmethanofuran dehydrogenase subunit B; *fwd*C means formylmethanofuran dehydrogenase subunit C; *fwd*D means formylmethanofuran dehydrogenase subunit D; *fwd*H means formylmethanofuran dehydrogenase subunit H; *fwd*F means formylmethanofuran dehydrogenase subunit F; *fwd*G means formylmethanofuran dehydrogenase subunit G; *ftr* means formylmethanofuran--tetrahydromethanopterin N-formyltransferase; *mtd* means methylenetetrahydromethanopterin dehydrogenase; *mer* means 5,10-methylenetetrahydromethanopterin reductase)

# Text S1 The analyzed method for collected data

The Raw Data was downloaded in NCBI database (Jinqi Biotechnology Co., Ltd., Zhejiang, China). Then, these Raw Data processing using Trimmomatic was conducted to acquire the Clean Data for subsequent analysis. To the samples taken from complex environment, such as water, soil and so on, MEGAHIT could be used to assemble the Clean Data. All samples’ Clean Data are compared to each Scaffolds respectively by MEGAHIT to acquire the PE reads not used. All the reads not used in the forward step of all samples are combined and then use the software of MEGAHIT for mixed assembly with the parameters same as single assembly. Filter the fragment shorter than 500 bp in all of Scaftigs for statistical analysis. The Scaftigs (≥ 500 bp) assembled from both single and mixed are all predicted the ORF by MetaGeneMark, and filtered the length information shorter than 90 nt from the predicted result with default parameters. The CD-HIT is adopted to remove redundancy and obtain the unique initial gene catalogue (the genes here refers to the nucleotide sequences coded by unique and continuous genes), which is cluster by identity 95%, coverage 90%, and choose longest to the representative sequences. The clean data of each sample is mapped to initial gene catalogue using BBMAP software and get the number of reads to which genes mapped in each sample. Based on the number of mapped reads and the length of gene, statistic the abundance information of each gene in each sample.

# Text S2 Extracellular polymeric substances extraction

The sludge (8 mL) was mixed with 0.05% NaCl solution (6 mL). The mixture was centrifuged at 4000 rpm for 5 min and discard the supernatant. The sludge was mixed with 0.05% NaCl solution again. The mixture was then sequentially vortexed for 5 min, heated in a water bath at 65 ^o^C for 2 min and centrifuged at 4000 rpm for 15 min. The supernatant obtained was filtered with 0.45 µm membranes to become the loosely bound EPS (L-EPS). The sludge mixed with 0.05% NaCl solution was sequentially vortexed for 15 min, heated in a water bath at 65 ^o^C for 30 min and centrifuged with 10000 rpm for 20 min. The supernatant obtained was filtered with 0.45 µm membranes was regarded as the tightly bound EPS (T-EPS).

# Text S3 Detection of typical redox mediators

Two sets of batch tests (with/without QS enhancement) were conducted in bottles fed with propanol and butanol to extract these redox mediators. The working volume of each bottle was 200 mL with 50 mL headspace. First, 180 mL simulated nutrient salt was added to the system, and propanol (equal to 0.5 gCOD) and butanol (equal to 1 gCOD) were added into systems. Moreover, the exogenous AHLs were also introduced into system, and its concentration was as same as Section 2.2. Then, all the systems were flushed with N_2_ for 5 min to eliminate oxygen dissolved in water. Next, 20 g inoculum was inoculated into each system, and the high purity N_2_ was used to eliminate extra oxygen (entering system during operation). Finally, rubber stoppers were used to seal all the systems. The systems were incubated in a constant temperature water bath to maintain mesophilic condition (37 °C ± 1 °C) and the shaking rate was 120 rpm. All the tests were conducted in ternary.

After three cycles, the liquid supernatant was used to detect these redox mediators. The concentration of flavin adenine dinucleotide (FAD) was detected by UPLC equipped with a fluorescence detector (excitation wavelength: 450 nm and emission wavelength: 520 nm) and a C18 column. Acetone and 0.2% acetate (ratio of 23:77) were used as the mobile phase at a flow rate of 0.5 ml/min. The detection method for 2-amino-3-carboxy-1,4-naphthoquinone (ACNQ) was similar to that for FAD. The UPLC was equipped with a fluorescence detector (excitation wavelength: 450 nm and emission wavelength: 520 nm) and an ultraviolet detector (520 nm). The mobile phase was changed to acetone and 0.2% acetate (ratio of 50:50) with a flow rate of 0.5 ml/min. The concentration of phenazine was measured by UPLC equipped with an ultraviolet detector (254 nm). The mobile phase was water and methanol (10:90), and the flow rate was 0.8 ml/min.

# Text S4 Cyclic voltammetry analysis

Cyclic voltammetry (CV) was conducted using a single-chamber electrochemical cell (50 mL), and was measured in the potential range from −0.6 V to 0.9 V (vs. Ag/AgCl) with a scan rate of 50–200 mV/s. Equation was used to analyze the related redox reactions and the electron transfer rate constant in further. The working electrode was the carbon felt, the counter and reference electrodes were graphite slice and saturated Ag/AgCl electrode respectively inserted into the rubber plug with the airway. The PBS solution (1M) was injected as the electrolyte (40mL), Moreover, 5 mL Na_2_SO_4_ (1M) solution and 5 mL sludge were also added into electrochemical cell.

# Text S5 Microbial community and metagenome analysis

As for microbial community, the genomic DNA extracted by the FastDNA SPIN Kit for soil (Mpbio, USA.) based on manufacturer’s instructions. For bacterial community, the bacterial 16S rRNA genes were amplified using the universal bacterial primers 27F (5’-AGRGTTYGATYMTGGCTCAG-3’) and 1492R (5’-RGYTACCTTGTTACGACTT-3’). After electrophoresis, The PCR products were purified using the AMPure® PB beads (Pacifc Biosciences, USA) and quantified with Quantus™ Fluorometer (Promega, USA). Purified products were pooled in equimolar and DNA library was constructed using the SMRTbell® Express Template Prep Kit 2.0 (Pacifc Biosciences, CA, USA) according to PacBio's instructions. Purified SMRTbell libraries were sequenced on the Pacbio Sequel II System (Pacifc Biosciences, CA, USA) by Majorbio Bio-Pharm Technology Co. Ltd. (Shanghai, China). The optimized-CCS reads were de-noised using DADA2_CCS plugin in the Qiime2 (version 2020.2) pipeline with recommended parameters, which obtains single nucleotide resolution based on error profiles within samples (Bolyen et al., 2019). Deblur denoised sequences are usually called amplicon sequence variants (ASVs).

Concerning metagenome, total genomic DNA was extracted using the NEXTFLEX™ Rapid DNA-Seq Kit (Omega Bio-tek, USA.) according to manufacturer’s instructions. DNA extract was fragmented to an average size of about 400 bp using Covaris M220 (Gene Company Limited, China) for paired-end library construction. Paired-end library was constructed using NEXTFLEX Rapid DNA-Seq (Bioo Scientific, Austin, USA). Adapters containing the full complement of sequencing primer hybridization sites were ligated to the blunt-end of fragments. Paired-end sequencing was performed on Illumina at Majorbio Bio-Pharm Technology Co., Ltd. (Shanghai, China) using NovaSeq Reagent Kits/HiSeq X Reagent Kits according to the manufacturer’s instructions. The paired-end Illumina reads were trimmed of adaptors, and low-quality reads (length<50 bp or with a quality value <20 or having N bases) were removed by fastp. A non-redundant gene catalog was constructed using CD-HIT with 90% sequence identity and 90% coverage. Reads after quality control were mapped to the non-redundant gene catalog with 95% identity using SOAPaligner, and gene abundance in each sample was evaluated through Reads Per Kilobase Million (RPKM) method in this study.

The raw data that support the findings of this study are available from Tsinghua University but restrictions apply to the availability of these data, which were used under license for the current study, and so are not publicly available. Data are however available from the authors upon reasonable request and with permission of Tsinghua University.

# Text S6 The modified Gompertz equation

The modified Gompertz equation was used for kinetics analysis(Zhao et al 2021), which was expressed in Eq.1:

| P_CH4_(t)=P*exp(-exp($\frac{K*e*(\lambda-t)}{P}+1))$ | (1) |
| --- | --- |

*where P is the maximum biogas production potential (mL), t is the reaction time (h), K was the maximum production gas rate (mL/ h). K is defined as the tangent in the inflection point, λ is the lag phase (h).*

# Text S7 The calculation of mantel, network, and microbial community assembly

Mantel test was conducted to examine the relationship between methane production and the relative abundance of microbes. This test was conducted using the 'mantel-test' function within the ‘linkET’ package in R-project (4.2.2). Co-occurrence network analysis was conducted using network inference, utilizing Spearman's coefficient (r > 0.7) and a significance level of (p-value < 0.05). All network diagrams were visualized using Gephi v0.9.3. Moreover, the robustness of a network is defined as the proportion of the remaining species in this network after random or targeted node removal (Yuan et al 2021). To simulate random species removal, 50% of nodes were randomly removed, and the proportion of residue nodes was regarded as the network’s robustness. To further explore the fundamental mechanisms driving microbial community assembly (Zhou et al 2014), we calculated the stochastic ratio (ST value) and modified stochasticity ratio (MST value).

# Text S8 The electron transfer flux for interspecies hydrogen transfer

According to Fick's law, we calculated the electron transfer flux for IHT (Eq.1) (de Bok et al 2004, Zhao et al 2024a). The maximum driving force for H_2_ diffusion was calculated by subtracting the highest H_2_ concentration produced by syntrophs during the oxidation of propionate and butyrate ([H_2 Syn_], *^Δ^G_Syn_*=0) from the lowest H₂ concentration achieved by methanogens during bicarbonate reduction ([H_2 Met_], *^Δ^G_Met_*=0), as shown in Eq.2. To determine the maximum electron transfer flux, an interbacterial distance of 0.5 μm was assumed(Zhao et al 2024a), and surface area of the flocs were calculated by average particle size.

| *i*=*D_f_*·(*S_cell_*/*d*)·([H_2_]*_Syn_*-[H_2_]*_Met_*)·n·F | (1) |
| --- | --- |
| ^Δ^G=^Δ^G^0^+RTln($\frac{C^{c}\cdot D^{d}}{A^{a}\cdot B^{b}}$) | (2) |

*where i is electric current (A), Df is diffusion coefficient of H_2_, in water (4.5×10^−5^ cm^2^/s), S_cell_ is surface area of the cells, [H_2_]_Syn_ is highest H_2_ concentration produced by syntrophs, [H_2_]_Met_ is the lowest H₂ concentration achieved by methanogens, n is mol of electrons per mol of H_2_,(n=2), F is Faradays constant (96485.3383 C/mol).*

**Reference:**

Cheng J, Su X, Liu M, Lu Z, Xu J, He Y (2023). Simultaneous regulation of biocathodic γ-HCH dechlorination and CH4 production by tailoring the structure and function of biofilms based on quorum sensing. *Environmental Pollution* **335:** 122357.

de Bok FAM, Plugge CM, Stams AJM (2004). Interspecies electron transfer in methanogenic propionate degrading consortia. *Water Research* **38:** 1368-1375.

Huang W, Cheng X, Li Y, Feng Q, Wu Y, Luo J (2025). Signaling molecule alleviates inhibitory impacts of surfactant on methane production during sludge and food waste co-digestion: Insights of electron bifurcation and quorum sensing. *Journal of Hazardous Materials* **484:** 136810.

Lv L, Chen J, Wei Z, Hao P, Wang P, Liu X *et al* (2024). A new strategy for accelerating recovery of anaerobic granular sludge after low-temperature shock: In situ regulation of quorum sensing microorganisms embedded in polyvinyl alcohol sodium alginate. *Bioresource Technology* **401:** 130709.

Ping J, Liu J, Dong Y, Song W, Xie L, Song H (2024). Biochar inoculated with Rhodococcus biphenylivorans altered microecological regulation by promoting quorum sensing and electron transfer: Up-regulation of related genes and enhancement of phenol and ammonia degradation. *Bioresource Technology* **397:** 130498.

Yang J-H, Huang D-Q, Wu G-G, Han N-N, Fan N-S, Jin R-C (2025). Quorum sensing-mediated microecological homeostasis in anammox consortia. *Journal of Hazardous Materials* **492:** 138285.

Yuan MM, Guo X, Wu L, Zhang Y, Xiao N, Ning D *et al* (2021). Climate warming enhances microbial network complexity and stability. *Nature Climate Change* **11:** 343-348.

Zhang B, Xu R, Liang Y, Wei G, Wei C, Chen H *et al* (2024). Carrier with cyclodextrin and quorum sensing synergy: An efficient method for selective enrichment of anammox bacteria. *Chemical Engineering Journal* **481:** 148461.

Zhao D, Zhang P, Liu Y, Li Q, Wang G, Chen R *et al* (2024a). Biochar-mediated synergistic promotion of DIET and IHT: Kinetic and thermodynamic insights into propionate two-step syntrophic methanogenesis. *Chemical Engineering Journal* **486:** 150310.

Zhao S, Chen W, Luo W, Fang H, Lv H, Liu R *et al* (2021). Anaerobic co-digestion of chicken manure and cardboard waste: Focusing on methane production, microbial community analysis and energy evaluation. *Bioresource Technology* **321:** 124429.

Zhao S, Zhu S, Liu S, Song G, Zhao J, Liu R *et al* (2024b). Quorum Sensing Enhances Direct Interspecies Electron Transfer in Anaerobic Methane Production. *Environmental Science & Technology* **58:** 2891-2901.

Zhou J, Deng Y, Zhang P, Xue K, Liang Y, Van Nostrand JD *et al* (2014). Stochasticity, succession, and environmental perturbations in a fluidic ecosystem. *Proceedings of the National Academy of Sciences* **111:** E836-E845.

Zhu Q, Qian D, Yuan M, Li Z, Xu Z, Liang S *et al* (2023). Revealing the roles of chemical communication in restoring the formation and electroactivity of electrogenic biofilm under electrical signaling disruption. *Water Research* **243:** 120421.

1. *Corresponding author: School of Environment, Tsinghua University, 30^#^ Haidian Shuangqing Road, 100084, China. Email address: [rpliu@tsinghua.edu.cn](mailto:rpliu@tsinghua.edu.cn) [↑](#footnote-ref-1)
